# Phenotypic variability in bread wheat root systems at the early vegetative stage

**DOI:** 10.1186/s12870-020-02390-8

**Published:** 2020-04-28

**Authors:** Yinglong Chen, Jairo Palta, P. V. Vara Prasad, Kadambot H. M. Siddique

**Affiliations:** 1 The UWA Institute of Agriculture, and School of Agriculture and Environment, The University of Western Australia, LB 5005, Perth, WA 6001 Australia; 2 CSIRO Agriculture & Food, Private Bag No. 5, Wembley, WA 6913 Australia; 3 Department of Agronomy, Kansas State University, Manhattan, Kansas, 66506 USA

**Keywords:** Bread wheat, Root phenomics, Root system morphology, Root distribution

## Abstract

**Background:**

Understanding root system morphology in bread wheat is critical for identifying root traits to breed cultivars with improved resource uptake and better adaptation to adverse environments. Variability in root morphological traits at early vegetative stages was examined among 184 bread wheat genotypes originating from 37 countries grown in a semi-hydroponic phenotyping system.

**Results:**

At the onset of tillering (Z2.1, 35 days after transplanting), plants had up to 42 cm in shoot height and 158 cm long in root depth. Phenotypic variation existed for both shoot and root traits, with a maximal 4.3-fold difference in total root length and 5-fold difference in root dry mass among the 184 genotypes. Of the 41 measured traits, 24 root traits and four shoot traits had larger coefficients of variation (CV ≥ 0.25). Strong positive correlations were identified for some key root traits (i.e., root mass, root length, and these parameters at different depths) and shoot traits (i.e., shoot mass and tiller number) (*P* ≤ 0.05). The selected 25 global traits (at whole-plant level) contributed to one of the five principal components (eigenvalues> 1) capturing 83.0% of the total variability across genotypes. Agglomerative hierarchical clustering analysis separated the 184 genotypes into four (at a rescaled distance of 15) or seven (at a rescaled distance of 10) major groups based on the same set of root traits. Strong relationships between performance traits (dry mass) with several functional traits such as specific root length, root length intensity and root tissue density suggest their linkage to plant growth and fitness strategies.

**Conclusions:**

Large phenotypic variability in root system morphology in wheat genotypes was observed at the tillering stage using established semi-hydroponic phenotyping techniques. Phenotypic differences in and trait correlations among some interesting root traits may be considered for breeding wheat cultivars with efficient water acquisition and better adaptation to abiotic stress.

## Background

Bread wheat (*Triticum aestivum*) is a staple crop in many countries, being one of the most economically and socially important cereal crops for human food and animal feed. Efforts have been made to identify the constraints to crop growth and yield, with increasing interest in breeding wheat cultivars with suitable root systems that possess desirable root traits for efficient utilization of resources [1–3]. Root systems play a critical role in the uptake of water and nutrients, and often are the first plant organ that senses and responds to various edaphic stresses, such as soil water deficit, salinity, waterlogging and nutrient deficiencies.

Plant traits, either performance traits or functional traits, are widely accepted as good proxies to represent plant strategies in ecology and agriculture [4, 5]. According to Violle et al. (2007), performance traits contribute directly to fitness due to their effects on growth, reproduction and survival, while functional traits are those morpho-physio-phenological traits that have an impact on performance traits and thus indirectly on fitness [4]. The identification of root performance or functional traits conferring efficiency in resource acquisition and adaptation to edaphic stresses, particularly in drying soil environments, has increased in research and breeding programs. Drought stress, particularly end-of-season (i.e. terminal) drought, is a major constraint to wheat yield and stability in arid and semiarid regions of the world [6–8]. Root system morphological traits, referred to as root phenes, include root length, numbers, angles, density at various depths, and root length in different diameter classes [9]. Studies on root phenomics rely on high-throughput phenotyping pipelines to measure root properties of hundreds of genotypes of the same or different crop species [10]. However, wide-scale use of root genetic information in breeding and selection programs remains a challenge [11]. This is primarily due to the difficulty in phenotyping root traits efficiently and accurately, and observing root traits in soil, especially under field conditions [12, 13]. Several phenotyping methods have been used to evaluate roots, including hydroponic systems using growth pouches (or germination paper) [12, 14, 15], aeroponic and agar-plate systems [16], soil-filled rhizotron [3, 17–19], and deep column techniques [20]. Current wheat root phenotyping studies are limited to the very early seedling stage, with only a few phenotyping platforms are used for measuring whole root systems in wheat, such as the germination paper technique [12, 21] and clear pots [22]. Soil-filled PVC tubes (columns) and glass-walled rhizoboxes are commonly used in root studies, but these techniques require extensive labor input and large space when phenotyping large sets of genotypes [20, 23, 24]. A semi-hydroponic phenotyping system [25] was developed to characterize root trait variability from a core collection of narrow-leafed lupin (*Lupinus angustifolius* L.) [26, 27]. This platform has been used in root phenotyping studies in other crops, such as chickpea (*Cicer arietinum* L.) [28], maize (*Zea mays* L) [29], barley (*Hordeum vulgare* L.) and soybean (*Glycine max* (L.) Merr.) (Chen et al. unpublished), but has not been applied to wheat.

Some studies have evaluated phenology and morphological traits in large collections of wheat genetic resources [30, 31]. Current worldwide wheat diversity is divided according to European and Asian origins [32, 33]. Here we aimed to characterize the phenotypic variability in root morphology in bread wheat in the selected 184 accessions from various resources and determine selection criteria for wheat genotypes with root traits for efficient acquisition of soil resources and adaptation to edaphic stresses. This study aimed to characterize phenotypic variability in root system traits in a set of wheat germplasms at the early growth stage (tillering) and determine the relationship among shoot and root traits and between plant performance and functional traits in relation to plant strategies in resource acquisition. A semi-hydroponic phenotyping system [25] was used to measure detailed root system characteristics and trait-trait correlation among 184 genotypes of bread wheat.

## Results

### Phenotypic variation in early shoot and root growth

**Fig. 1:**
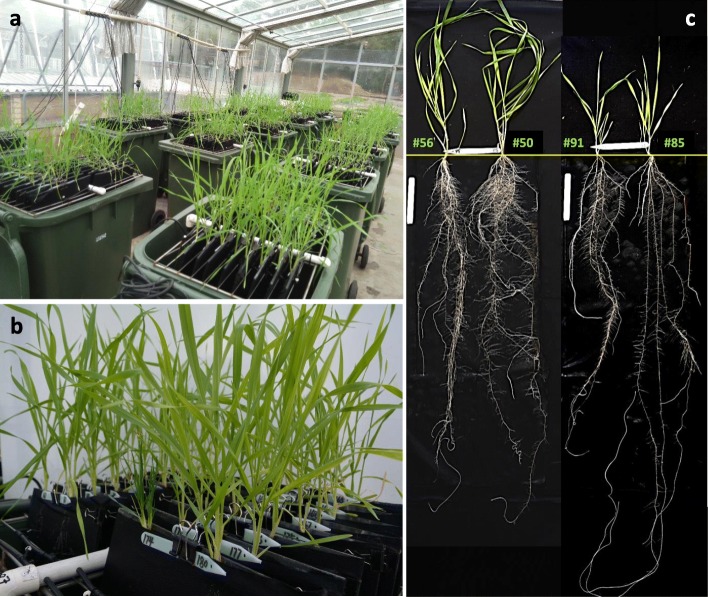
**a** Wheat plants grew in a semi-hydroponic system in a glasshouse, **b** a close shot of plants in the bin, and (**c**) example plants of four wheat genotypes with contrasting shoot and root system morphology grown in the semi-hydroponic phenotyping platform in a temperature-controlled glasshouse 35 days after transplanting. Genotypes (left to right): #56 (Chyamtang), #50 (Kulung), #91 (Compton, USA), and #85 (Horoshiri-Komugi). Bar = 10 cm

**Fig. 2:**
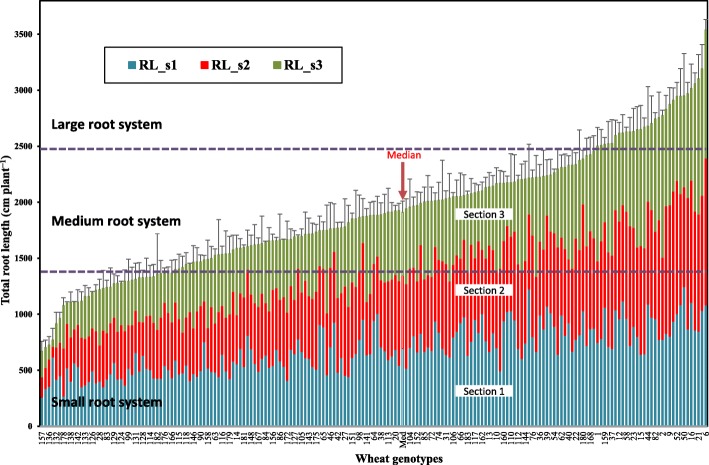
Cumulative barplot showing phenotypic variation in root length among 184 wheat genotypes grown in a semi-hydroponic phenotyping platform 35 days after transplanting. Data were plotted from the lowest to the highest total root length (RL) values. For each genotype, root lengths for the three sections (depths from the top of the root system) were plotted in three different colors. RL_s1: total root length in section 1 (0–20 cm; blue bars); RL_s2: total root length in section 2 (20–40 cm, red bars); RL_s3: total root length in section 3 (below 40 cm, green bars). The median value for all genotypes is presented (unfilled bars). Genotypes were divided into three groups with small, medium or large root systems with the medium interval defined as the median value of RL ± the standard deviation. The upper and lower boundaries of the medium interval were constructed by adding to, or subtracting from, the median point. The error bar for each genotype was based on the total root length

At 35 days after transplanting (DAT), wheat plants were up to 42 cm tall (i.e. genotype #55) (Fig. 1b) and the longest seminal roots up to 158 cm long (#73) with a root growth rate ranging from 1.2 cm (#119) to 4.4 cm per day (#73) (Table 1; mean data with standard errors for each genotype are not presented but available upon request). Significant differences (*P* < 0.01) in shoot growth (e.g. shoot height, leaf number and tiller number) and rooting and branching pattern (root length, density, depth etc.) were observed among genotypes. Total root length per root system (RL) ranged from 670 to 3538 cm with a median value of 1937 cm (Table 1). Among the 184 genotypes, six genotypes had RL < 1000 cm per plant, 79 genotypes had RL values from 1000 to 2000 cm per plant, and five genotypes had RL > 3000 cm (Fig. 2). Based on the median value (i.e. 1937 cm plant^− 1^) ± standard deviation (552 cm plant^− 1^), 37 genotypes had small root systems (RL < 1385 cm plant^− 1^) and 31 genotypes had large root system (RL > 2489 cm plant^− 1^). The Russian-origin genotype Hopea (#6) had the longest RL (3538 ± 188 cm) and greatest shoot mass (450 mg) per plant, which was 4- and 9-fold higher than Australian genotype Tincurrin (#157), respectively (Table 1; Fig. 2). For each root depth along the root profile (layer), root length varied greatly among genotypes, and for some genotypes, the proportion of root length among the three depths differed significantly (Fig. 2). Maximal root depth of the longest root-system genotype Ferrugineum was 2.5-times than that of the shallowest root-system genotype W7984. Root length density ranged from 0.47 to 2.47 with a median value of 1.35 cm cm^− 2^. Root length intensity varied about 3-fold among the genotypes (Table S1). The root length ratio between section 1 (top 0–20 cm layer) and the rest of the roots (RLR_s1/sub) ranged from 0.3 to 3.81, indicating different root distributions and root morphological patterns between various root depths and tested genotypes (Table 1).

**Table 1:** Descriptive statistics of 25 important traits (22 root traits, and four shoot traits) in 184 wheat genotypes grown in a semi-hydroponic phenotyping platform

Trait	Abbreviation	Minimum	Maximum	Mean	Median	Std. deviation	CV	Significance
Seminal root length zone1	SRLZ1	27.7	120	85.3	86.3	14.4	0.17	**0.000**
Seminal root length zone2	SRLZ2	6.8	37.7	24.1	24.6	5.66	0.23	**0.000**
Maximal root depth	MRD	44.3	158	109	111	18.8	0.17	**0.000**
Seminal and primary root number	SRN	5.67	48.3	11.1	10.8	3.21	**0.29**	0.258
Total root length	RL	670	3538	1902	1937	552	**0.29**	**0.000**
Root diameter	RD	0.26	0.48	0.31	0.30	0.02	0.07	0.052
Specific root length	SRL	60.5	172.7	122.3	121.4	17.8	0.15	**0.000**
Root length Intensity	RLI	8.12	31.1	17.8	17.6	4.55	**0.26**	**0.000**
Root tissue density	RTD	75.4	175	117	118	17.5	0.15	0.334
Root length s1	RL_s1	260	1245	687	694	213	**0.31**	**0.000**
Root diameter s1	RD_s1	0.26	0.40	0.30	0.29	0.02	0.07	0.939
Root length s2	RL_s2	93.9	1313	652	653	216	**0.33**	**0.000**
Root diameter s2	RD_s2	0.23	0.43	0.28	0.27	0.03	0.09	**0.001**
Root length s3	RL_s3	59.3	1266	563	562	227	**0.40**	**0.000**
Root diameter s3	RD_s3	0.29	0.69	0.35	0.35	0.04	0.12	**0.000**
Root length in sub-root layer	RL_sub	153	2455	1215	1220	409	**0.34**	**0.000**
Root diameter in sub-root layer	RD_sub	0.27	0.56	0.31	0.31	0.03	0.10	**0.000**
Root length ratio	RLR_s1/sub	0.30	3.81	0.65	0.63	0.32	**0.49**	**0.000**
Root mass	RM	50.7	305	159	155	53.1	**0.33**	**0.000**
Shoot mass	SM	47.5	450	230	221	81.9	**0.36**	**0.000**
Total dry mass	TDM	98.2	975	390	379	166	**0.43**	**0.000**
Root mass ratio	RMR	0.45	1.12	0.74	0.73	0.15	0.20	**0.000**
Shoot height	SH	8.87	42.7	21.9	22.0	5.85	**0.27**	**0.000**
Leaf number	LN	3.67	35.0	8.50	8.15	3.11	**0.37**	**0.000**
Tiller number	TN	1.00	5.33	2.40	2.37	0.84	**0.35**	**0.000**

Fourteen of 25 Traits with CVs (coefficients of variation) ≥0.25 appear in red and bold type. Probability (*P*) values were based on a GLM multivariate analysis of 184 genotypes (see Table 3 for units of each trait, and Table S1 for the data of additional 16 root traits)

Example wheat plants with large variation in both shoots and root morphological traits are shown in Fig. 1c. The two plants of similar shoot size on the left side of the image had totally different root systems: genotype #050 (Kulung) had remarkably more lateral roots and wider root angles with higher root density in the top 20 cm layer than its partner genotype #065 (Chyamtang) and the other two genotypes in the figure. In contrast, #085 (Horoshiri-Komugi) developed much deeper primary and lateral roots with fewer seminal (and primary) roots than the other three plants (Fig. 1c).

Average root diameter per genotype ranged from 0.26 to 0.48 mm (Table 1), and 84.5% of the total root length was in root diameter range between 0.05 and 0.45 mm (Fig. S1). Some 68% of the total root length was in the 0.5–0.15 mm root diameter class. Root diameter length in the three root sections followed a similar trend to the total root diameter class length (DCL). Roots thicker than 0.75 mm were mostly in the upper layer (0–20 cm), accounting for approximately 2.4% of total root length. The tested genotypes differed significantly in specific root length (SRL) (*P* < 0.001, Table 1). SRL ranged from 60.5 (#119: W7984) to 172.7 (#128: Stiletto) with a median value of 121.4 m g^−^^1^ (Fig. S2). Cultivars Tincurrin, Sunco, Centurk, Cotipora, Krichauff, Yitpi and Harper were in the genotype group with the highest SRL values indicating a root system with more fine roots.

The 184 genotypes were ranked for each trait based on the mean values; the top 20 and bottom 20 genotypes for total root length are presented in Table S2. Of the top 20 genotypes for total root length, 17 also ranked in the top 20 for RL_sub (root length in the section below 20 cm) (excluding Kulung, Flint and Weibullsholm Jo 3045), and 14 for root mass (excluding Austro Bankut, Nachipundo, College Eclipse, Cotipora, Rongotea and Pitic 62), ten for shoot mass, and six for root growth. Of the bottom 20 genotypes for root length, 13 also ranked in the bottom 20 for RL_s1 and root mass, 15 for RL_sub, 11 for root growth rate, and nine for shoot mass (Table S2). Among the 20 small-root-system genotypes, 11 were identified as slow root growers with their root growth rate ranked among the lowest, and nine also had their shoot mass ranked in the lower 20. Interestingly, several recently released Australian cultivars, including Hydra, Scepter, Impress CI Plus, Harper and Trojan were in the lowest 20 genotype group with low values for root size (root length and root mass).

### General variation and correlation among traits

Most of the 40 measured traits differed significantly among genotypes except for seminal and primary root number, root diameter, root tissue density (root mass per root volume) and root diameter in section 1 (*P* < 0.01) (Tables 1 and S3). Twenty-four root traits and four shoot traits had coefficients of variation (CV) values > 0.25. Root morphological traits, such as root length, root length density, root length intensity (root length per depth), root area, and root volume had relatively larger variation among genotypes. These traits also varied between genotypes in each root section (depth). However, relatively less variation existed for some of the root traits including maximal root depth (longest seminal root length), root diameter, and specific root length, as indicated by low CV values.

**Fig. 3:**
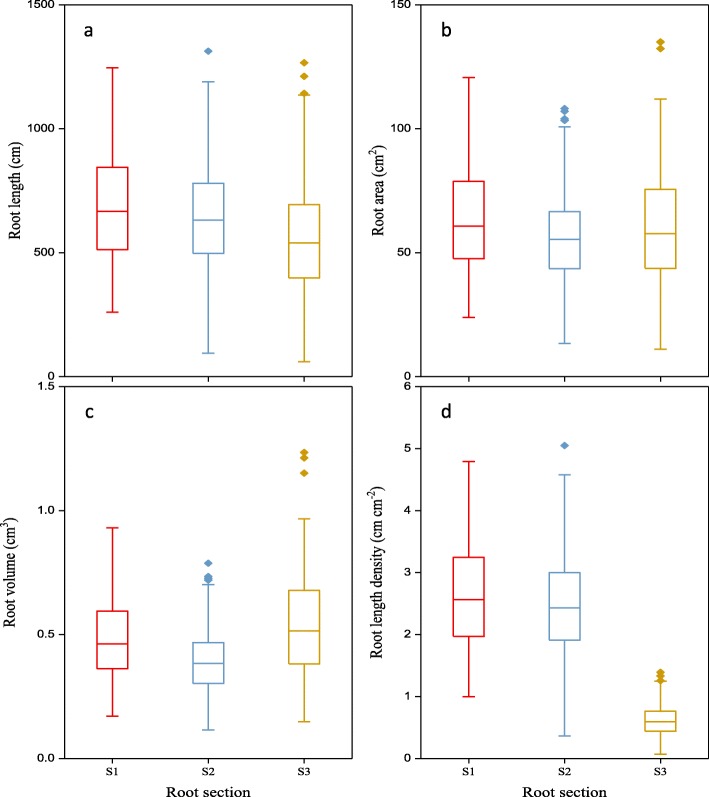
Variation in root morphological traits in the three root sections among 184 wheat genotypes grown in a semi-hydroponic phenotyping platform 35 days after transplanting. Genotype data were combined for each section. The boxplots were confined to the first and third quartiles with the middle lines being the median. Four root traits were plotted: (**a**) root length, (**b**) root surface area, (**c**) root volume, and (**d**) root length density. Three root sections: s1, 0–20 cm layer; s2, 20–40 cm layer; and s3, 40–110 cm layer

For root traits at the various root depths, small variation in root length, root surface area, and root volume occurred among the three root sections across all genotypes (Fig. 3). Average root length in s1 (0–20 cm layer), s2 (20–40 cm layer) and s3 (below 40 cm layer) were 687, 652 and 563 cm, respectively (Table 1). Of the three root sections, s2 had relatively smaller root area and root volume, while s1 and s2 had significantly more root length density than in s3 (2.64 and 2.51 vs. 0.62 cm cm^− 2^; Fig. 3).

When considering the origin of the genotype, large variations existed between genotypes from different countries for most of the measured traits (Figs. S3, S4). For example, mean root length by country ranged from 703 cm per plant in genotypes from Zimbabwe to 3191 cm per plant in genotypes from Algeria. Average root mass by country ranged from 76 mg in genotypes from Zimbabwe to 298 mg in genotypes from Italy, and shoot mass ranged from 137 mg in genotypes from Armenia to 420 mg in genotypes from Algeria.

**Fig. 4:**
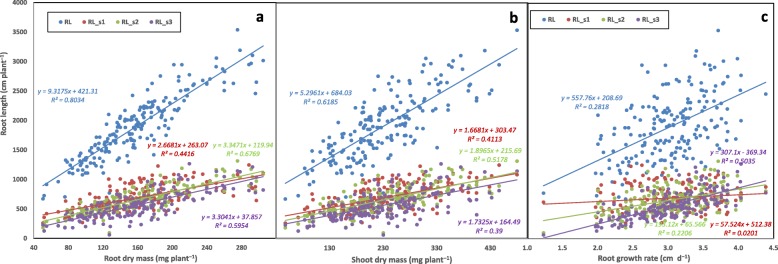
Correlations between (**a**) root length and root dry mass, (**b**) root length and shoot dry mass, and (**c**) root length and root growth rate in 184 wheat genotypes grown in a semi-hydroponic phenotyping platform 35 days after transplanting. Total root length (RL) and root length in three depths (RL_s1, RL_s2 and RL_s3) were used in the analyses

**Fig. 5:**
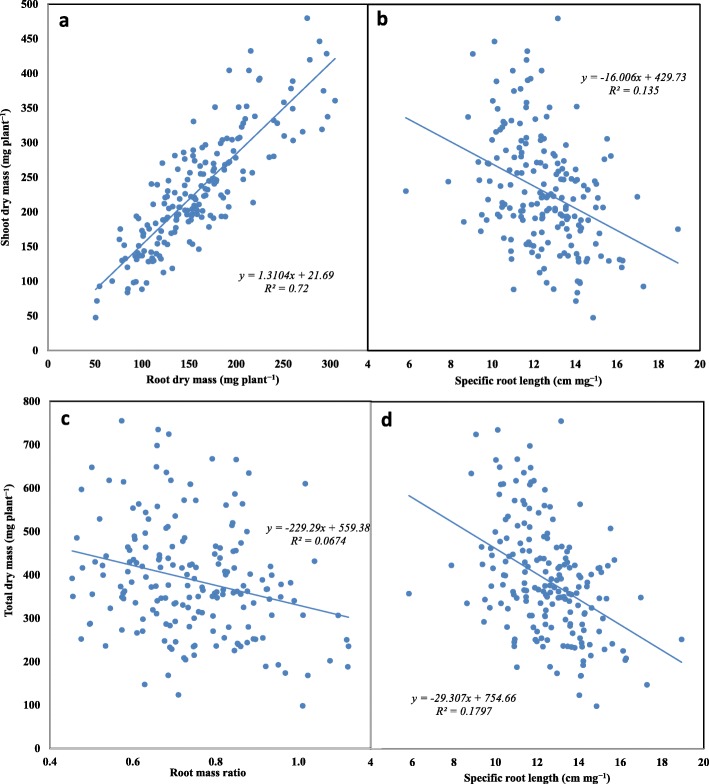
Correlations (**a**) between shoot dry mass and root dry mass, (**b**) shoot dry mass and specific root length, (**c**) total dry mass and root mass ratio, and (**d**) total dry mass and specific root length in 184 wheat genotypes grown in a semi-hydroponic phenotyping platform 35 days after transplanting (all *P* < 0.01)

A set of 25 traits, including four shoot traits were used to establish the Pearson correlation matrix to identify correlations among the measured traits (Table S4). Most of the selected traits had strong correlation with other traits (*P* < 0.01). All root traits were highly correlated with shoot dry mass and total dry mass (except root diameter (RD) and RD_s1 (root diameter in section 1) and leaf number (LN) and tiller number (TN) (except seminal and primary root number (SRN) and RD_s1) (mostly *P* < 0.01). Root length (RL) of the total root system and root sections and root length intensity (RLI) were strongly associated with shoot height (SH) (*P* < 0.05; Table S4). RL was significantly correlated with all other traits except specific root length (SRL), root tissue density (RTD), RD_s1, and root mass ratio (RMR) (mostly *P* < 0.01). For example, RL was strongly correlated with root mass (RM) (*R*^*2*^ = 0.80, *P* < 0.01; Fig. 4a), shoot mass (SM) (*R*^*2*^ = 0.62, *P* < 0.01; Fig. 4b) and root growth ratio (RGR) (*R*^*2*^ = 0.28, *P* < 0.05; Fig. 4c). RL in each section also had significant associations with RM, SM and RGR, respectively (*P* < 0.01) (Fig. 4, Table S4). SM was strongly associated with RM (*R*^*2*^ = 0.72, *P* < 0.01; Fig. 5a; Table S4). SRL was negatively correlated with SM and total dry mass (*P* < 0.01; Fig. 5b, d). Root mass ratio had a negative correlation with total dry mass (*P* < 0.01; Fig. 5c). Maximal root depth (MRD) had a strong correlation with RM, SM, total dry mass (TDM), LN and TN, respectively, but not SH (*P* < 0.05; Table S4). SRN was correlated with SH, but not LN or TN, and TN strongly correlated with LN (*P* < 0.01; Table S4). SRL had a significant association with RTD, RD in section 1 and section 3, RM, SM, TDM, LN and TN (all *P* < 0.01 except *P* < 0.05 for LN; Table S4).

### Determination of trait variation

**Fig. 6:**
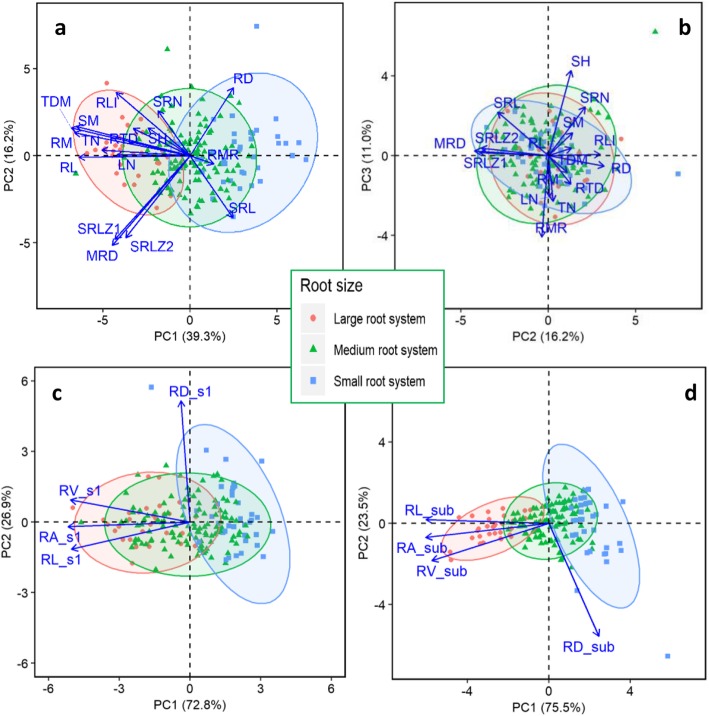
Principal component analysis of (**a**, **b**) 16 selected global traits (12 root-related and four shoot traits) and (**c**) four root traits in the top section (0–20 cm) and (**d**) sub-section (below 20 cm) with genotypes presented by root system size, among 184 wheat genotypes grown in a semi-hydroponic phenotyping platform 35 days after transplanting. The position of each global trait is shown for (**a**) PC1 vs. PC2 representing 55.5% of the variability, and (**b**) PC2 vs. PC3 representing 27.2% of the variability. The position of each (**c**) top section trait and (**b**) sub-section trait for PC1 vs. PC2 representing 100% of the variability, respectively

**Fig. 7:**
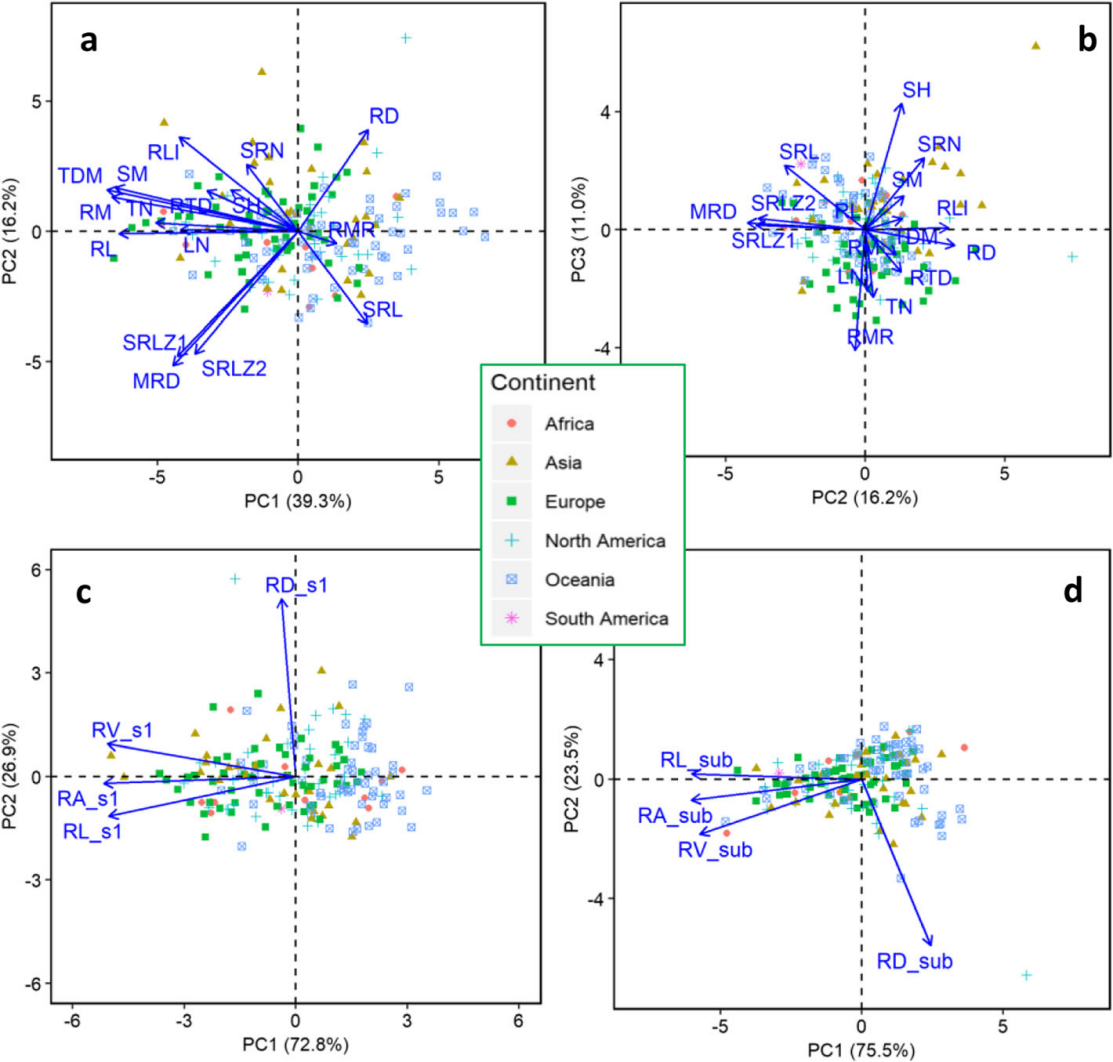
Principal component analysis of (**a**, **b**) 16 selected global traits (12 root-related and four shoot traits) and (**c**) four root traits in top section (0–20 cm) and (**d**) sub-section (below 20 cm), with genotypes presented by continent of origin, among 184 wheat genotypes grown in a semi-hydroponic phenotyping platform 35 days after transplanting. The position of each global trait is shown for (**a**) PC1 vs. PC2 representing 55.5% of the variability, and (**b**) PC2 vs. PC3 representing 27.2% of the variability. The position of each (**c**) top section trait and (**d**) sub section trait for PC1 vs. PC2 representing 100% of the variability

Principal component analysis (PCA) was performed for (1) global traits (Table 2; Figs. 6a, b, 7a, b), (2) top section traits (Figs. 6c, 7c), and (3) sub section traits (Fig. 6d, 7d). The first PCA on16 selected global traits (excluding mathematically linked traits, such as root length density, root volume and root area at whole root system and in root sections) revealed five principal components (PCs) with eigenvalues > 1, which captured 83.0% of the variability across the tested genotypes (Table 2). PC1 accounted for 39.3% of the total variability, consisting mostly of whole plant traits such as RL, RLI, RM, SM, TDM, LN and TN. PC2 accounted for 16.3% of the total variation primarily for seminal root length zone 1 (SRLZ1), seminal root length zone 1 (SRLZ2), MRD, and RD. PC3 (11.0% variability) consisted of shoot height (SH), seminal and primary root number (SRN) and root mass ration (RMR). The PCA on four local traits each contained two PCs with PC1 accounting for 96.9% (top section; Figs. 6c, 7c) and 75.5% (sub section; Figs. 6d, 7d). Genotypes are presented by root size (Fig. 6) or continent of origin (Fig. 7). The biplots exhibited a clear separation of genotypes in three root-size categories showing positive contribution of all global traits to root system size except SRL, RD and RMR (Fig. 6a). Of the global traits, RL, RM, SM, TDM and MRD contributed the most to root size. Three of the four local traits (except RD) in both top and sub sections were strongly associated with root system size (Fig. 6c, d). As for continents, most of the global traits tended to have a greater influence on genotypes with European origin than those from Oceania (Fig. 7a). This was also the case for top section traits (Fig. 7c), but not sub section traits (Fig. 7d).

**Table 2:** Variable loading scores of 16 selected global traits (12 root-related and four shoot traits) and the proportion of variation of each principal component

Trait	PC1	PC2	PC3	PC4	PC5
SRLZ1	0.60	**0.67**	0.03	−0.21	0.21
SRLZ2	0.52	**0.66**	0.06	−0.07	0.06
MRD	0.62	**0.72**	0.04	−0.18	0.18
SRN	0.26	−0.36	**0.42**	−0.25	0.09
RL	**0.89**	0.02	0.03	0.37	0.20
RD	−0.34	**−0.53**	−0.09	−0.02	0.36
SRL	−0.34	0.50	0.38	**0.65**	−0.14
RLI	**0.59**	−0.50	0.01	0.55	0.10
RTD	0.46	−0.22	−0.25	**−0.69**	−0.19
RM	**0.93**	− 0.18	− 0.15	0.05	0.25
SM	**0.91**	−0.24	0.20	− 0.02	− 0.08
TDM	**0.95**	−0.22	0.06	0.01	0.05
RMR	−0.19	0.07	**−0.70**	0.14	0.54
SH	0.34	−0.22	**0.73**	−0.09	0.13
LN	**0.59**	−0.01	−0.35	0.14	−0.55
TN	**0.71**	−0.05	−0.39	0.23	−0.28
Variation proportion					
Eigenvalue	6.28	2.57	1.75	1.57	1.08
Variance (%)	39.3	16.2	11.0	9.8	6.7
Cumulative variability (%)	39.3	55.5	66.5	76.3	83.0

Rotation converged in 25 iterations using Varimax with Kaiser normalization. For each trait, the large variable loading score crossing the six components appears in bold. Three principal components with eigenvalues > 1 were extracted and considered significant (Tabachnik and Fidell, 1996)

### Identification of groups among the genotypes based on root characters

*K*-means clustering analysis identified five relatively homogeneous groups of genotypes based on the nine root-related traits with CV values > 0.25 (Table S5). Cluster center data for each group of genotypes identified all root traits except SRN contributing significantly to group separation (*P* < 0.01). The number of genotypes in the five groups ranged from 16 to 56, indicating variation in the degree of homogeneity among tested genotypes. Cluster #3 grouped 16 genotypes with the highest values of cluster centers in all root traits except root length ratio (RLR_s1/sub). Cluster #1 contained 32 genotypes, with the lowest values for all root traits, apart from the largest value for RLR_s1/sub. Clusters 2, 4, and 5 consisted of 28, 56 and 52 genotypes, respectively, with moderate values for cluster centers. The dendrogram of agglomerative hierarchical clustering (AHC) separated the 184 genotypes into four major clades at a rescaled distance of 15 using the average linkage method with squared Euclidean distance as the interval measurement on the same set of 24 root traits (Fig. S5). The genotypes were further separated into seven groups when a rescaled distance of 10 was used. Representative genotypes from each group were selected for further studies. In terms of country, the number of genotypes in each country varied among the 37 countries, from 1 genotype to 55 genotypes. Australia (55 genotypes), Mexico (18), Nepal (13) and Russia (10) are the top four countries having 10 or more genotypes included in this study. The 55 genotypes from Australia were clustered into five groups (Fig. S4), with the majority of genotypes (34 genotypes, 61.8% of 55) in G7, followed by G6 (13, 23.6%), G4 and G3 (3 each), and G1 (2). However, three genotypes (#1, #74 and #148) originated from China were assigned in three different groups. These results demonstrated that genotypes from the same country were not always clustered into the same or closer group(s).

## Discussion

### Characterizing root trait variability and its implications for wheat breeding

There is an increasing interest in studying root phenomics, which is considered central to crop breeding [11, 34]. Among the measured root traits, root depth, total root length, root mass and root length density (RLD) at various depths, are most important for water and nutrient acquisition [17, 35]. Wheat selection and breeding programs have focused on aboveground traits and yield, with their impact on root traits often ignored [36]. The genotypes Hopea, Bahatans 87 and Ghurka ranked in the top 20 genotypes for root length with their significantly larger root systems (in terms of root length and root mass) and fast root growth rates (Table S2, Fig. 2). The vigorous root system of these wheat genotypes, although having better uptake of nitrogen in deep sandy soils when grown under current rainfall [37, 38], can also prompt to a premature terminal drought, as their shallow root system can use the soil available water in the topsoil layers very fast. The advantage of their massive shallow root system is likely the use of soil water from small rainfalls after flowering [37, 39]. Vigorous root system genotypes often have better crop establishment and shoot development and when their root system is deep (high vigorous), and improve water-use efficiency and grain yield in rainfed environments [40]. Several Australian commercial cultivars, including Drysdale, Harper, and Mace, were among the bottom 20 genotypes for total root length and root mass, indicating small root systems (Table S2). Two advanced breeding lines, IGW-3119 and IGW-3262, putatively adapted to dryland environments [41] developed moderate-size root systems as reflected in their root depth, root length and dry mass (Table 1). Modern wheat cultivars with smaller root systems than older cultivars have been confirmed in other studies [13, 42, 43], as an unintended result of breeding for increasing grain yield. Selection for wheat grain yield in dry environments and dry seasons has focused mainly in reducing phenology, particularly time to anthesis to evade the severe effects of drought on grain filling [40]. The high yielding of modern wheat cultivars is related to a short time to anthesis and the short time to anthesis to a small root system [44]. The wheat root system grows through allocation of the daily photosynthetic carbon. The allocation is 42–48% from leaf emergence (Z11) to floral initiation (double ridge, Z31) at which stage it abruptly decreases to 18% and subsequently to 4% by booting (Z27) [45, 46]. Consequently, wheat cultivars with short time to anthesis have less time for root growth and hence smaller root system size than those with longer time to anthesis [44]. Genotypes with vigorous root growth and greater root length, root mass, and RLD in early growth stages are often better able to capture water, facilitate crop establishment and shoot development, and improve grain yield under rainfed agriculture systems [47, 48].

Root depth is an important trait in association with moisture and nutrient uptake at depth. Our phenotyping study showed a three-fold difference in rooting depth among the tested wheat genotypes (Table 1). A positive correlation between deep rooting during early growth and early root vigor benefits crop establishment for improved photosynthetic capacity and biomass production [49, 50]. Rice genotypes with deep-rooting properties improved drought tolerance and nitrogen accumulation, and increased harvest index and cytokinin flux from roots to shoots during grain filling [51]. The capacity for vigorous growth and deep rooting enables access to subsoil water and better photosynthesis, and grain filling under drought conditions, and thus contributes to drought avoidance [8]. Genotypes with deep root systems are better suited to drought-prone areas [35, 52] and are beneficial for the capture of subsoil nitrate in maize [1].

Considerable variation in root diameter and root length in diameter classes among genotypes, indicated by specific root length (SRL, calculated as root length divided by root mass) was observed in this study (Figs. S1, S2). SRL is a widely used trait for describing root phenomics, demonstrating how root mass can be used for nutrient acquisition. It can characterize economic aspects of the root system and may indicate environmental changes [53]. Some wheat genotypes, including the Australian cultivars Bonnie Rock, Cranbrook, Cobra, Drysdale, Frame, Kennedy, Livingston and Yitpi, had high SRL indicating thinner roots, which presumably increased the surface area per unit root volume, improving water- and nutrient-uptake efficiencies [41]. Moreover, genotypes with small root diameters (i.e., larger SRL) and large root length densities at depth are better adapted to drought conditions [6, 54]. In northern New South Wales and southern Queensland in Australia, where wheat is grown in winter and spring on stored soil water from the previous summer rainfall, and terminal drought often occurs, genotypes with more fine roots and larger SRL are expected to save water in the subsoil for use during grain filling. This is mainly because the narrow metaxylem vessels of thin seminal roots have low root hydraulic conductivity [6]. High root length density deep in the soil profile is a critical trait for accessing deep soil water after anthesis, contributing to high grain yield under terminal drought [48, 55–57]. An evaluation of old, modern and newly released cultivars of winter wheat under drought conditions in China ratified that moderate drought conditions stimulated root growth in newly released cultivars enabling access to deep soil water and leading to higher grain yields [42]. Therefore, genotypes with root properties such as small diameters, greater specific root lengths, and increased root length densities in the subsoil and root hair densities, can be selected as candidate parents.

This study measured 43 traits including 39 root-related traits and four shoot traits, under the categories of “performance traits” and “functional traits”. According to Violle et al. (2007) [4], performance traits are direct measurements of plant fitness, such as shoot and root biomass, and functional traits are those parameters having an indirect impact on plant fitness through their effects on growth, reproduction and survival. The Pearson correlation matrix identified strong correlations between performance traits and some functional traits (*P* < 0.01; Figs. 4, 5; Table S4). Evidence of strong correlations between plant dry mass (shoot, root and total) and several functional traits such as specific root length (SRL), root length intensity (RLI) and root tissue density (RTD) demonstrate that suites of correlated root traits are linked to plant growth strategies [58, 59]. Studies have shown that fast-growing plants tend to have high SRL and root nitrogen concentration in root tissue, and often have high respiration rates in fine roots, which most likely reflects metabolic activity related to nutritional uptake and assimilation [59, 60].

Genotypes with similar root system size (root length and root mass) may distribute differently in the soil profile, as measured by root length density, a mirror trait of root length (Figs. 3, 6, 7). Several recent studies in maize demonstrated that genotypes with reduced lateral root branching density, steeper root growth angles, and reduced production of crown roots had better root growth deeper in the profile, nitrogen uptake and yield in infertile soil (reviewed in Lynch 2019) [58]. Genotypes from different geographic origin showed some variations (Fig. S3, S4) reflecting potential ecological and climatic linkages that require further exploration.

Our observations of early growth in wheat plants also showed that some genotypes did not have close relationships between root system size and shoot performance, such as those illustrated in Fig. 1c, demonstrating that variation in shoot height does not always reflect root system size, and vice versa. Analyses of genetic variability for root traits in a Spring Wheat Association Mapping Panel revealed weak negative relationships between plant height and root dry weight [36]. In wheat several studies have reported conflicting results regarding to the relationship between root traits and plant height [13, 61]. In this study, leaf area was not measured due to the feasibility of handling large number of plants and the primary focus of assessing root-related traits at the tillering stage. More shoot-related traits such as phenological development and shoot morphological and physiological traits could be measured in future studies.

### Validation of root characters in soil and at later growth stages

Using the established semi-hydroponic phenotyping system [25], this study and others [20, 22, 28, 29, 56], elucidated phenotypic variability in numerous root morphological traits among 184 genotypes of bread wheat originating from 37 countries. Phenotypic variations in these root phenomics are likely to be associated with differences among genotypes in their capacity to respond to environments to optimize resources acquisition [62]. Attempts have been made to incorporate digital root images acquired in the semi-hydroponic system, automated high-throughput computing, and collaboration platforms, such as DIRT [63] to analyze crop root phenomics, particularly the quantification of root growth angles.

Five genotypes with putative differences in root system size were selected in a follow-up validation experiment using 24-L rhizoboxes (24 cm × 10 cm, 100 cm deep) filled with farm soil [24]. The experiment confirmed that phenotypic variation in root system size at the onset of tillering (Z2.1, 35 DAT) in this phenotyping study was reproducible at the booting stage (Z4.9; 63 days after sowing) in the validation experiment [24]. The positive correlation between root growth at the early vegetative and late ontogenetic stages and the consistent ranking of genotypes in some important root traits demonstrates the capacity of the semi-hydroponic phenotyping system to provide simple, relevant growing conditions for root phenotyping studies. A series of early studies in wild narrow-leafed lupin also identified the liability of the semi-hydroponic phenotyping system via repeated experiments under various environments including the field [26, 60, 64]. Several studies have investigated root phenotyping correlations under controlled and field environments. For example, significant root trait correlations were found between wheat seedlings at the two-leaf stage grown on moist germination paper and in the field [56]. Nevertheless, there is a limitation of translating the phenotyping data acquired from the semi-hydroponic system in the early growth stage into wheat breeding programs. Phenotyping systems, therefore, need to produce reliable rankings of root traits, and the choice of growth media needs to be carefully considered. The development of modern root imaging technologies and root simulation modeling could be incorporated into phenotyping platforms.

## Conclusions

Using the established semi-hydroponic phenotyping platform, this study characterized the root properties of 184 genotypes of bread wheat and discovered large variation in several root traits and observed correlations between performance traits and functional traits at the onset of tillering. Genotypes with interesting root phenomics, such as large root length and mass, high root length density at depth, deep roots and thin roots, and trait−trait relationships, could be used for further evaluation in soil and under various growing conditions, and crossing with widely adapted cultivars in breeding programs. Furthermore, such valuable root traits could be incorporated into marker-assisted selection using recent advances in genome editing technologies and sequencing data in the International Wheat Genome Sequencing Consortium for breeding cultivars with improved drought tolerance and resource use efficiency.

## Methods

### Plant material and experimental design

A set of 184 genotypes of bread wheat from 37 countries of origin were selected for this study. The set included 99 genotypes from a core collection of 372 accessions representing the worldwide wheat diversity generated by two sets of SSR markers and deposited at the French National Institute for Agricultural Research’s Clermont-Ferrand Genetic Resources Center (http://www.clermont.inra.fr/umr-asp) [33]. The INRA cereal germplasm collection consists of > 10,000 accessions of hexaploid wheat with some 3000 accessions originating from France, Europe and the rest of the world [32]. The set also included the top 10 varieties by area grown in Western Australia in 2014 and some selected Australian cultivars released in various years. Therefore, the collection used in this study contains a significant proportion of the world’s genetic resources of bread wheat, making it a useful tool for future studies of wheat genetic diversity.

Seeds of 184 genotypes of bread wheat were sourced from Australian Grains Genebank, Australian Grain Technologies, Australia Winter Cereals Collection (AWCC), CSIRO and InterGrain. The list of wheat genotypes included in this phenotyping study is in Table S6 including their country of origin and seed providers.

Plants were grown in an established semi-hydroponic phenotyping system described elsewhere [25]. Briefly, this system consists of a 240-L plastic wheelie bin (top 75 × 58 cm, height 108 cm), 20 growth units made of a 5 mm thick acrylic panel (260 × 480 mm) wrapped in a black calico cloth, and an automatically controlled irrigation system.

Each bin system was filled with 30 L solution containing (in m M): K (1220), P (20), S (1802), Ca (600), Mg (200), Cu (0.2), Zn (0.75), Mn (0.75), B (5), Co (0.2), Na (0.06), Mo (0.03), Fe (20) and N (2000). Plants grew in the units between the panel and cloths moistened via the pumping system during the growth period. Plants were grown in a temperature-controlled glasshouse at The University of Western Australia, Perth (31°58′ S, 115°49′ E), with a day/night temperatures of 22/16 °C, mean relative humidity of 66% and midday maximum photosynthetic photon flux density of 1440 μmol photons m^− 2^ s^− 1^ for the duration of the study (April–May 2016)(Fig. 1a). A randomized block design was used consisting of 184 wheat genotypes and four replicates. The four replicate plants of each genotype were grown in four different bins. Five bins accommodating 200 plants (including one plant of each genotype, plus 16 buffer plants) were considered as one replicate (40 plants per bin, and two plants per growth unit / glass-cloth panel with 2 cm distance between inter panels). The 40 plants in each bin were randomly planted. There were 30–50 cm gap between bins to allow access to each bin and avoiding competitions of plants between bins. Each bin fitted with two wheels at the bottom for ease of moving around and repositioning weekly to minimize the environmental impact during the experiment. The glasshouse was maintained under routine hygiene practices and no pesticides were applied.

### Plant growth

Wheat seeds were surface sterilized and sown in multiple-welled plastic seedling trays filled with washed river sand (< 2 mm) for germination. Five days after sowing, when the seedlings roots were approx. 2–3 cm long, uniform seedlings were carefully removed from the tray, washed with deionized (DI) water, and transplanted into the growth glass-cloth pouches of the semi-hydroponic system.

### Sampling and measurements

Plants were harvested 35 days after transplanting (DAT) when plants were at the onset of tillering (Z2.1; Zadoks’ scale of cereal growth). At harvest, shoot height, leaf and tiller number per plant were manually recorded. Shoot height was measured with a ruler from the surface of the soil to the tip of the tallest leaf. The growth panels were taken out of the bin and laid on a flat bench. The cloth was removed to expose root systems for photographing using a photographing system installed above the top of the bench with a fluorescence light and camera (Nikon D5200). After photographing, shoots were separated by cutting the shoots from the roots at the crown. The longest seminal root length at the branching (with lateral roots) and non-branching (without any lateral roots) zones were measured manually at harvest (Table 3). The length of the deepest primary and/or lateral root of each plant was defined as the maximal root depth, which is the sum of root length at the branching zone and root length at non-branching zone. Seminal root number (including primary roots) was counted and recorded for each plant. Subsamples of roots were collected for morphological and architectural measurements by cutting the root system into 20-cm sections starting from the base and optically scanned before drying (see below). Shoot and roots were then dried in an air-forced oven at 65 °C for 72 h to determine shoot and root dry masses.

**Table 3:** Description of 38 root-related traits and four shoot traits in 184 wheat genotypes characterized in a semi-hydroponic phenotyping system

Trait	Abbreviation	Description	Unit
Seminal root length zone1	SRLZ1	The longest seminal root length at branching zone (Z1)	cm
Seminal root length zone2	SRLZ2	The longest seminal root length at non-branching zone (Z2)	cm
Maximal root depth	MRD	The longest seminal root length (i.e., root depth, Z1 + Z2)	cm
Seminal and primary root number	SRN	Seminal and primary root number (from seeds)	number per plant
Total root length	RL	Total root length per plant	cm
Root diameter	RD	Average root diameter	mm
Total root area	RA	Root surface area	cm^2^
Total root volume	RV	Root volume	cm^3^
Root length density	RLD	Root length per unit area (0–110 cm depth, 1430 cm^2^)	cm cm^−2^
Specific root length	SRL	Total root length per unit root dry mass	m g^−1^ dry mass
Root length Intensity	RLI	Total root length per unit root depth	cm cm^− 1^
Root tissue density	RTD	Root dry mass per unit root volume	mg cm^−3^
Diameter class length	DCL	Root length within a diameter class	mm
Relative diameter class length	rDCL	Root diameter class length/Total root length	%
Root length s1	RL_s1	Total root length in section 1 (s1, 0–20 cm; top-root layer)	cm
Root diameter s1	RD_s1	Average root diameter in section 1 (s1, 0–20 cm)	mm
Root area s1	RA_s1	Total root surface area in section 1 (s1, 0–20 cm)	cm^2^
Root volume s1	RV_s1	Total root volume in section 1 (s1, 0–20 cm)	cm^3^
Root length density s1	RLD_s1	Root length per unit area in section 1 (s1, 260 cm^2^)	cm cm^−2^
Root length s2	RL_s2	Total root length in section 2 (s2, 20–40 cm)	cm
Root diameter s2	RD_s2	Average root diameter in section 2 (s2, 20–40 cm)	mm
Root area s2	RA_s2	Total root surface area in section 2 (s2, 20–40 cm)	cm^2^
Root volume s2	RV_s2	Total root volume in section 2 (s2, 20–40 cm)	cm^3^
Root length density s2	RLD_s2	Root length per unit area in section 2 (s2, 260 cm^2^)	cm cm^−2^
Root length s3	RL_s3	Total root length in section 3 (s3, 40–110 cm)	cm
Root diameter s3	RD_s3	Average root diameter in section 3 (s3, 40–110 cm)	mm
Root area s3	RA_s3	Total root surface area in section 3 (s3, 40–110 cm)	cm^2^
Root volume s3	RV_s3	Total root volume in section 3 (s3, 40–110 cm)	cm^3^
Root length density s3	RLD_s3	Root length per unit area in section 3 (s3, 910 cm^2^)	cm cm^−2^
Root length in sub-root layer	RL_sub	Combined root length in sub-root layer (s2 & s3)	cm
Root diameter in sub-root layer	RD_sub	Combined average root diameter in sub-root layer (s2 & s3)	mm
Root area in sub-root layer	RA_sub	Total root surface area in sub-root layer (s2 & s3)	cm^2^
Root volume in sub-root layer	RV_sub	Total root volume in sub-root layer (s2 & s3)	cm^3^
Root length density in sub-root layer	RLD_sub	Root length per unit area in sub-root layer (s2 & s3)	cm cm^−2^
Root length ratio	RLR_s1/sub	Root length in section 1 (top-root layer) over sub-root layer	
Root growth rate	RGR	Average daily root growth (based on the longest seminal or primary root growth at 35 days after seed sowing)	cm d^−1^
Root mass	RM	Root dry mass	mg
Shoot mass	SM	Shoot dry mass	mg
Total dry mass	TDM	Total dry mass (sum of root and shoot dry mass)	mg
Root mass ratio	RMR	Root-to-shoot dry mass ratio	
Shoot height	SH	Shoot height measured to the tallest leaf	cm
Leaf number	LN	Number of leaves per plant	
Tiller number	TN	Number of tillers per plant	

Root subsamples were scanned in greyscale at 300 dpi using a desktop scanner (Epson Perfection V700, Long Beach, CA, USA). Root images were analyzed in WinRHIZO (Prof v2009, Regent Instruments, Montreal, QC, Canada) to acquire root length, root area, root volume, average root diameter and root length in diameter class for each 20-cm root section. Imaging analyses used the debris removal filter of discounting objects less than 1 mm^2^ with a length-width ratio of < 8 in the image. The roots were partitioned into ten diameter classes at 0.1 mm intervals except for 0.05 mm intervals in the two lower classes.

### Root trait measurements and calculations

Root morphological traits of each root system (global level), including root length, root surface area, root volume, average root diameter, and diameter class length (DCL, root length within a diameter class) were calculated from the respective local traits per 20-cm section computed by WinRHIZO from scanned root images (Table 3). The first 20-cm root section (s1) and the section beyond 40 cm (to 110 cm) were considered the ‘top-root layer’ and ‘sub-root layer’ sections, respectively. At harvest, the length of the longest seminal root of each plant divided by the number of days of growth (35 days) was referred to root growth rate. Root length density (RLD) was calculated as total root length per unit area since roots were grown in a two-dimensional unit (root areas were 1430, 260, 260 and 910 cm^2^ for the entire root system, s1, s2 and s3, respectively). Root length intensity was defined as the total root length divided by root depth. Specific root length (SRL, root length per unit root mass) was used as an indicator of root thickness. Relative diameter class length (rDCL) equals DCL divided by root length yielding a proportion of root length to normalize the disparity between plants of different sizes. Detailed descriptions of the 42 root traits and four shoot-related traits are in Table 3.

### Statistical analysis

General Linear Model (GLM) multivariate analysis was performed for genotype and plant position main effects using SPSS Statistics (Version 19, IBM, USA). Data of four replications of each genotype were subjected to the analyses. No significant differences between plants grown on the edge and in the middle of the bins were identified for all measured traits across genotypes (Table S3). Constant multivariate standard errors of skewness (0.18) and kurtosis (0.36) indicated no serious departure from multivariate normality when all parameters were included in the GLM analysis. General correlations between each trait pair of 25 traits (Table 1) were included in the Pearson correlation analysis, and correlations were considered statistically significant when *P* ≤ 0.05. Mathematically linked traits such as root area, root volume, root length density and root growth rate at both global and local/section levels were excluded in the correlation analysis. The same set of traits was also used for principal component analysis (PCA) for both global (whole root system) and local (section) traits to identify determinants of root morphological variability across genotypes [65]. The relationship between genotypes from six continents and three root-size categories was also shown on the biplots produced in R. Genotypes were divided into three groups having small (37 genotypes), medium (116) or large (31) root systems based on total root length per plant (RL). The medium root-sized group interval was defined as the median value of RL (i.e., 1937 cm plant^− 1^) ± standard deviation (552 cm plant^− 1^). The upper and lower boundaries of the medium interval were constructed by adding to, or subtracting from, the median point. Hierarchical cluster analysis was used to determine variance among nine root traits of the above set of traits (excluded four shoot traits) and homogeneous groups among genotypes using the average linkage method with squared Euclidean distance as the interval measurement. Cluster centers of five identified groups were generated by *K*-means clustering analysis. Selected key root traits at various root depths and root traits of genotypes from different countries were plotted as grouped box charts, respectively using the Origin graphing package (OriginLab, Northampton, Massachusetts, USA). The boxes were based on median values of the defined traits by the first and third quartiles, and individual data points that fell outside the whiskers (1.5 times the interquartile range from the median) were considered as outliers. Other line graphs were produced in Excel 2013 using the mean data generated from SPSS Statistics.

## Supplementary information

**Additional file 1: Table S1.** Descriptive statistics of additional 16 root traits (root area, root volume and root length density of the whole root system and root sections, and root growth rate) in 184 wheat genotypes grown in a semi-hydroponic phenotyping platform. **Table S2.** Wheat genotypes ranked in the top or bottom 20 genotypes for total root length (RL) of the 184 genotypes, some of which were also ranked in the top or bottom 20 for other traits. **Table S3.** General Linear Model (GLM) multivariate analysis for plant position as the main effect in selected root traits (shoot height, root mass and shoot mass). **Table S4.** Pearson’s correlation matrix for 25 traits (including 21 root traits and four shoot traits) in 184 wheat genotypes. **Table S5.** Mean values (cluster centers) of five groups generated by K-Means clustering analysis for nine root-related traits in 184 wheat genotypes. **Table S6.** The184 genotypes of wheat (Triticum aestivum) from 37 countries of origin used in this study. **Figure S1.** Root diameter class length (DCL, cm) in sections and relative diameter class length (rDCL, %) among 184 wheat genotypes grown in a semi-hydroponic phenotyping platform 35 days after transplanting. Percentage values for rDCL in each diameter class are plotted on the secondary axis. Mean DCL values in each root section are presented with SEs for the total root length in the respective root diameter class. **Figure S2.** Phenotypic variation in specific root length (SRL) among 184 wheat genotypes grown in a semi-hydroponic phenotyping platform 35 days after transplanting. Data were plotted from the lowest to the highest SRL values. The median value for all genotypes is presented (red bar). **Figure S3.** Variation among the 37 countries of origin in (**a**) total root length, (**b**) shoot and root dry mass (SM and RM, respectively) in 184 wheat genotypes grown in a semi-hydroponic phenotyping platform 35 days after transplanting. Root data are the means for each country. Total root length in section 1 (RL_s1, 0–20 cm), section 2 (RL_s2, 20–40 cm), and section 3 (RL_s3, 40–110 cm) ± SE of total root length of all sections is presented. Country names are ordered by total root length value. The number of genotypes in each country varied and ranged from one to 55 (see Table S6). **Figure S4.** Variation among the 37 countries of origin in (**a**) total root length, (**b**) root length density, (**c**) root length intensity, (**d**) root length ratio, (**e**) root dry mass, and (**f**) shoot dry mass in 184 wheat genotypes grown in a semi-hydroponic phenotyping platform 35 days after transplanting. Country names are ordered by the median values of root length from least to most. The boxplots were confined to the first and third quartiles with the middle lines being the median. The number of genotypes in each country varied and ranged from one to 55 (see Table S6). **Figure S5.** Dendrogram of agglomerative hierarchical clustering (AHC) using the average linkage method with squared Euclidean distance as the interval measurement on 19 selected root traits with CVs ≥0.25. The 184 wheat genotypes were assigned to one of four general clades (Clade I, II, III or IV) at a rescaled distance of 15 (left dashed line) containing seven groups (G1 to G7) at a rescaled distance of 10 (right dashed line). See Table S6 for a list of the 184 wheat genotypes used in this study.

## Data Availability

All data generated or analyzed during this study are included in this published article and its supplementary information files.

## Abbreviations

AHC: Agglomerative hierarchical clustering
AWCC: Australia Winter Cereals Collection
CSIRO: Australian Commonwealth Scientific and Industrial Research
CV: Coefficients of variation
DAT: Days after transplanting
DI: Deionized
DIRT: Digital imaging of root traits
GLM: General Linear Model
P: Probability
PC: Principal component
PCA: Principal component analysis
PVC: Polyvinyl chloride
R2: R-squared (the coefficient of determination)

## Description of the following 42 plant traits is given in Table 3

SRLZ1: Seminal root length zone1
SRLZ2: Seminal root length zone2
MRD: Maximal root depth
SRN: Seminal and primary root number
RL: Total root length
RD: Root diameter
RA: Total root area
RV: Total root volume
RLD: Root length density
SRL: Specific root length
RLI: Root length Intensity
RTD: Root tissue density
DCL: Diameter class length
rDCL: Relative diameter class length
RL_s1: Root length s1
RD_s1: Root diameter s1
RA_s1: Root area s1
RV_s1: Root volume s1
RLD_s1: Root length density s1
RL_s2: Root length s2
RD_s2: Root diameter s2
RA_s2: Root area s2
RV_s2: Root volume s2
RLD_s2: Root length density s2
RL_s3: Root length s3
RD_s3: Root diameter s3
RA_s3: Root area s3
RV_s3: Root volume s3
RLD_s3: Root length density s3
RL_sub: Root length in sub-root layer
RD_sub: Root diameter in sub-root layer
RA_sub: Root area in sub-root layer
RV_sub: Root volume in sub-root layer
RLD_sub: Root length density in sub-root layer
RLR_s1/sub: Root length ratio
RGR: Root growth rate
RM: Root mass
SM: Shoot mass
TDM: Total dry mass
RMR: Root mass ratio
SH: Shoot height
LN: Leaf number
TN: Tiller number
