# Supplementary material for: Phenotypic variability in bread wheat root systems at the early vegetative stage
Source: BMC Plant Biol. 2020 Apr 28;20:185. doi: 10.1186/s12870-020-02390-8 (PMC7189723; doi:10.1186/s12870-020-02390-8)
Supplement: Supplementary file 1 — Additional file 1: Table S1. Descriptive statistics of additional 16 root traits (root area, root volume and root length density of the whole root system and root sections, and root growth rate) in 184 wheat genotypes grown in a semi-hydroponic phenotyping platform. Table S2. Wheat genotypes ranked in the top or bottom 20 genotypes for total root length (RL) of the 184 genotypes, some of which were also ranked in the top or bottom 20 for other traits. Table S3. General Linear Model (GLM) multivariate analysis for plant position as the main effect in selected root traits (shoot height, root mass and shoot mass). Table S4. Pearson’s correlation matrix for 25 traits (including 21 root traits and four shoot traits) in 184 wheat genotypes. Table S5. Mean values (cluster centers) of five groups generated by K-Means clustering analysis for nine root-related traits in 184 wheat genotypes. Table S6. The184 genotypes of wheat (Triticum aestivum) from 37 countries of origin used in this study. Figure S1. Root diameter class length (DCL, cm) in sections and relative diameter class length (rDCL, %) among 184 wheat genotypes grown in a semi-hydroponic phenotyping platform 35 days after transplanting. Percentage values for rDCL in each diameter class are plotted on the secondary axis. Mean DCL values in each root section are presented with SEs for the total root length in the respective root diameter class. Figure S2. Phenotypic variation in specific root length (SRL) among 184 wheat genotypes grown in a semi-hydroponic phenotyping platform 35 days after transplanting. Data were plotted from the lowest to the highest SRL values. The median value for all genotypes is presented (red bar). Figure S3. Variation among the 37 countries of origin in (a) total root length, (b) shoot and root dry mass (SM and RM, respectively) in 184 wheat genotypes grown in a semi-hydroponic phenotyping platform 35 days after transplanting. Root data are the means for each country. Total root length in se [file 12870_2020_2390_MOESM1_ESM.pdf]

Table S1. Descriptive statistics of additional 16 root traits (root area, root volume and root length density of the whole root system and of root sections, and root growth rate) in 184 wheat genotypes grown in a semi-hydroponic phenotyping platform.

Trait	Abbreviation	Minimum	Maximum	Mean	Median	Std. deviation	CV	Significance
Total root area	RA	65.7	319	181	182	50.8	**0.28**	**0.000**
Total root volume	RV	0.50	2.59	1.40	1.36	0.39	**0.28**	**0.000**
Root length density	RLD	0.47	2.47	1.33	1.35	0.39	**0.29**	**0.000**
Root area s1	RA_s1	23.9	121	63.8	63.9	19.5	**0.31**	**0.000**
Root volume s1	RV_s1	0.17	0.93	0.48	0.47	0.15	**0.31**	**0.000**
Root length density s1	RLD_s1	1.00	4.79	2.64	2.67	0.82	**0.31**	**0.000**
Root area s2	RA_s2	13.4	108	56.2	55.6	17.9	**0.32**	**0.000**
Root volume s2	RV_s2	0.12	0.79	0.39	0.38	0.12	**0.31**	**0.000**
Root length density s2	RLD_s2	0.36	5.05	2.51	2.51	0.83	**0.33**	**0.000**
Root area s3	RA_s3	11.0	135	61.1	60.3	23.4	**0.38**	**0.000**
Root volume s3	RV_s3	0.15	1.23	0.54	0.51	0.20	**0.37**	**0.000**
Root length density s3	RLD_s3	0.07	1.39	0.62	0.62	0.25	**0.40**	**0.000**
Root area in sub-root layer	RA_sub	24.4	229	117	117	37.8	**0.32**	**0.000**
Root volume in sub-root layer	RV_sub	0.26	1.95	0.93	0.90	0.29	**0.31**	**0.000**
Root length density in sub-root layer	RLD_sub	0.43	6.30	3.13	3.13	1.02	**0.33**	**0.000**
Root growth rate	RGR	1.23	4.39	3.04	3.07	0.53	0.17	**0.000**

All the 16 root traits except RGR had CVs (coefficients of variation) ≥0.25 (appeared in red and bold type). Probability (*P*) values were based on a GLM multivariate analysis of 184 genotypes (see Table 3 for units of each trait).

Table S2. Wheat genotypes ranked in the top or bottom 20 genotypes for total root length (RL) of the 184 genotypes, some of which were also ranked in the top or bottom 20 for other traits.

Code #	Genotype name	RL	RL_s1	RL_sub	RGR	RM	SM
**6**	Hopea	♥	♥	♥	♥	♥	♥
**41**	Bahatans 87	♥	♥	♥		♥	♥
**21**	Ghurka	♥		♥		♥	♥
**34**	Seu Seun 27	♥		♥	♥	♥	
**16**	Ardito	♥	♥	♥	♥	♥	
**25**	Orlandi	♥		♥		♥	
**50**	Kulung	♥	♥			♥	♥
**71**	Austro Bankut	♥	♥	♥			
**52**	Nachipundo	♥	♥	♥			♥
**35**	Blueboy	♥		♥		♥	♥
**9**	Redman	♥		♥		♥	♥
**92**	Corin	♥		♥		♥	
**2**	College Eclipse	♥		♥	♥		♥
**59**	Cotipora	♥		♥	♥		
**82**	Rongotea	♥		♥			
**4**	Flint	♥				♥	
**44**	Weibullsholm Jo 3045	♥	♥			♥	
**8**	Pitic 62	♥		♥			
**15**	Arawa	♥		♥	♥	♥	♥
**26**	Blondynka	♥		♥		♥	♥
**Genotypes ranked in the lower 20 for root length (RL)**							
**123**	Cobra	♦		♦	♦	♦	♦
**83**	Buck Buck S	♦	♦			♦	
**177**	Machete	♦	♦				
**28**	Richelle	♦	♦				
**75**	Songlen	♦	♦				
**126**	Mace	♦		♦	♦	♦	♦
**164**	Excalibar	♦	♦	♦	♦	♦	
**133**	Hydra	♦	♦	♦		♦	♦
**121**	Bonnie Rock	♦	♦	♦		♦	
**142**	Scepter	♦		♦	♦		♦
**96**	Turda 81	♦		♦	♦		
**138**	Drysdale	♦	♦	♦			
**134**	Impress Cl Plus	♦		♦	♦	♦	♦
**178**	Magsil 30	♦	♦			♦	
**139**	Grenade Cl Plus	♦		♦	♦	♦	♦
**132**	Harper	♦	♦	♦	♦	♦	♦
**119**	W7984	♦		♦	♦		
**136**	Trojan	♦	♦	♦	♦	♦	♦
**79**	Limpopo	♦	♦	♦	♦	♦	
**157**	Tincurrin	♦	♦	♦		♦	♦

Root length (RL), RL in top 20 cm section (RL_s1), RL in the section below 20 cm (RL_sub), root growth rate (RGR), root mass (RM), and shoot mass (SM).

Table S3. General Linear Model (GLM) multivariate analysis for plant position as a main effect in selected root traits (shoot height, root mass and shoot mass).

**Source**		**Type III Sum of Squares**	**DF**	**Mean Square**	***F***	**Sig.**
**Corrected Model**	Shoot height	2.21	1	2.20	0.032	0.858
	Root mass	11708	1	11708.01	2.621	0.106
	Shoot mass	16966	1	16966.49	1.416	0.235
**Plant position**	Shoot height	2	1	2.20	0.032	0.858
	Root mass	11708	1	11708.01	2.621	0.106
	Shoot mass	16966	1	16966.49	1.416	0.235
**Error**	Shoot height	37071	540	68.65		
	Root mass	2412327	540	4467.27		
	Shoot mass	6471773	540	11984.77		

Table S4 Pearson's correlation matrix for 25 traits (including 21 root traits and 4 shoot traits) in 184 wheat genotypes.

Trait	SRLZ2	MRD	SRN	RL	RD	SRL	RLI	RTD	RL_s1	RD_s1	RL_s2	RD_s2	RL_s3	RD_s3	RL_sub	RD_sub	RLR_s1/sub	RM	SM	TDM	RMR	SH	LN	TN
**SRLZ1**	**	**		**	**			*		**	**	**	**	**	**	**	**	**	**	**			**	**
**SRLZ2**		**		**	**				*	**	**	**	**	**	**	**	**	**	**	**			**	**
**MRD**				**	**			*		**	**	**	**	**	**	**	**	**	**	**			**	**
**SRN**				*		**	**	**	**									**	**	**	**	**		
**RL**					**		**		**		**	**	**	**	**	**	**	**	**	**		**	**	**
**RD**						**		**	*	**	**	**	**	**	**	**	**		*	*			**	*
**SRL**								**		**				**		*		**	**	**			*	**
**RLI**									**		**		**		**			**	**	**		**	**	**
**RTD**											*	**		**	*	**		**	**	**			**	**
**RL_s1**										*	**	*	**		**		**	**	**	**		**	**	**
**RD_s1**												**		**		**	**							
**RL_s2**												**	**	**	**	**	**	**	**	**		**	**	**
**RD_s2**													**	**	**	**	**	**	**	**	**	*	**	**
**RL_s3**														**	**	**	**	**	**	**		*	**	**
**RD_s3**															**	**	**	**	**	**			**	**
**RL_sub**																**	**	**	**	**		**	**	**
**RD_sub**																	**	**	**	**	*		**	**
**RLR_s1/sub**																		*		*				
**RM**																			**	**		**	**	**
**SM**																				**	**	**	**	**
**TDM**																					**	**	**	**
**RMR**																						**		
**SH**																								
**LN**																								**

, Correlation is significant at the 0.01 level (2-tailed); ***, Correlation is significant at the 0.05 level (2-tailed). Mathematically linked traits such as RA, RV, RLD and RGR were excluded in the correlation analysis. See Table 3 for the trait descriptions.

Table S5 Mean values (cluster centers) of five groups generated by *K*-Means clustering analysis for 9 root-related traits in 184 wheat genotypes.

Root traits	Cluster#1	Cluster#2	Cluster#3	Cluster#4	Cluster#5	*F*	*Sig.*
**SRN**	*9.94*	11.56	**11.81**	11.53	10.86	1.7	0.1430
**RL**	*1147*	2469	**2949**	2044	1585	495	0.0000
**RLI**	*13.0*	20.3	**24.2**	19.4	15.6	48.2	0.0000
**RL_s1**	*452*	824	**908**	795	573	50.3	0.0000
**RL_s2**	*383*	837	**1061**	697	542	162	0.0000
**RL_s3**	*312*	808	**979**	552	469	123	0.0000
**RL_sub**	*695*	1645	**2040**	1249	1011	323	0.0000
**RLR_s1/sub**	**0.80**	0.54	*0.48*	0.70	0.61	4.7	0.0010
**RM**	*96*	214	**255**	165	133	151	0.0000
**Members**	32	28	16	56	52		
**%**	17.4	15.2	8.7	30.4	28.3		

Analysis of variance *F* statistics was performed, and both *F* and *P* values for each variable are given. Traits with large *F* values provide the greatest separation among clusters. The number of clusters was set at 5 using the iterate and classify method. Maximum iterations were 10. For each trait, minimums are italicized and maximums are in bold type.

Table S6 The184 genotypes of wheat (*Triticum aestivum*) from 37 countries of origin used in this study.

No.	Name	Country of origin[1]	Seed provider	Notes
1	Chinese Spring	China	AGG	Balfourier *et al.* (2007)[2]
2	College Eclipse	Australia	AGG	Balfourier *et al.* (2007)
3	Daeraad	South Africa	AGG	Balfourier *et al.* (2007)
4	Flint	United States	AGG	Balfourier *et al.* (2007)
5	Gamenya A	Australia	AGG	Balfourier *et al.* (2007)
6	Hopea	Russia	AGG	Balfourier *et al.* (2007)
7	Mocha De Espriga Branca	Portugal	AGG	Balfourier *et al.* (2007)
8	Pitic 62	Mexico	AGG	Balfourier *et al.* (2007); Narayanan *et al.* (2014)[3]
9	Redman	Canada	AGG	Balfourier *et al.* (2007); Narayanan *et al.* (2014)
10	Sarrubra	Russia[3]	AGG	Balfourier *et al.* (2007)
11	Squareheads Master	United Kingdom	AGG	Balfourier *et al.* (2007)
12	Alma	United Kingdom	AGG	Balfourier *et al.* (2007)
13	Admonter Fruh	Austria	AGG	Balfourier *et al.* (2007)
14	Andes 56	Mexico	AGG	Balfourier *et al.* (2007); Narayanan *et al.* (2014)
15	Arawa	New Zealand	AGG	Balfourier *et al.* (2007)
16	Ardito	Italy	AGG	Balfourier *et al.* (2007)
17	Spear	Australia	CSIRO	Released 1984
18	Coronation	Canada	AGG	Balfourier *et al.* (2007)
19	Africa Mayo	Kenya	AGG	Balfourier *et al.* (2007)
20	Fronthatch	Kenya	AGG	Balfourier *et al.* (2007)
21	Ghurka	Australia	AGG	Balfourier *et al.* (2007)
22	Kitchener	Canada	AGG	Balfourier *et al.* (2007)
23	Little Club	United States	AGG	Balfourier *et al.* (2007); Narayanan *et al.* (2014)
24	Opal	Netherlands	AGG	Balfourier *et al.* (2007)
25	Orlandi	Italy	AGG	Balfourier *et al.* (2007)
26	Blondynka	Poland	AGG	
27	Lang	Australia	CSIRO	
28	Richelle	Tunisia	AGG	
29	Kenya 350	Kenya	AGG	Balfourier *et al.* (2007)
30	Stanley	Canada	AGG	Balfourier *et al.* (2007)
31	Pamucak	Turkey	AGG	Balfourier *et al.* (2007)
32	Aurore	Australia	AGG	Balfourier *et al.* (2007)
33	Gular	Australia	AGG	Balfourier *et al.* (2007)
34	Seu Seun 27	South Korea	AGG	Balfourier *et al.* (2007)
35	Blueboy	United States	AGG	Balfourier *et al.* (2007)
36	Lammas	United Kingdom	AGG	Balfourier *et al.* (2007)
37	Diana	Poland	AGG	
38	Tom Thumb	Mexico	AGG	Balfourier *et al.* (2007)
39	Berzataka	Russia[4]	AGG	Balfourier *et al.* (2007)
40	Ebro 3	Spain	AGG	Balfourier *et al.* (2007)
41	Bahatans 87	Algeria	AGG	Balfourier *et al.* (2007)
42	BT-2281	Tunisia	AGG	Balfourier *et al.* (2007)
43	Agatha	Canada	AGG	Balfourier *et al.* (2007)
44	Weibullsholm Jo 3045	Finland	AGG	Balfourier *et al.* (2007)
45	Chitlang	Nepal	AGG	
46	Dhoje	Nepal	AGG	Balfourier *et al.* (2007)
47	Chirkung	Nepal	AGG	
48	Benchung	Nepal	AGG	

**49**	Gudel	Nepal	AGG	
**50**	Kulung	Nepal	AGG	
**51**	Monjo	Nepal	AGG	
**52**	Nachipundo	Nepal	AGG	
**53**	Jubing	Nepal	AGG	
**54**	Junbesi	Nepal	AGG	
**55**	Ritak 1	Nepal	AGG	
**56**	Chyamtang	Nepal	AGG	
**57**	Honggaoan	Nepal	AGG	
**58**	Centurk	United States	AGG	Balfourier *et al.* (2007)
**59**	Cotipora	Brazil	AGG	Balfourier *et al.* (2007)
**60**	Candeal De Arevalo	Portugal	AGG	Balfourier *et al.* (2007)
**61**	Dolis Puri	Russia[3]	AGG	Balfourier *et al.* (2007)
**62**	Buck Atlantico	Argentina	AGG	Balfourier *et al.* (2007)
**63**	Pato	Argentina	AGG	Balfourier *et al.* (2007)
**64**	Vakka	Finland	AGG	Balfourier *et al.* (2007)
**65**	Glenlea	Canada	AGG	Balfourier *et al.* (2007)
**66**	Matraderecskei	Hungary	AGG	Balfourier *et al.* (2007)
**67**	Chanate	Mexico	AGG	Balfourier *et al.* (2007)
**68**	Barani 70	Pakistan	AGG	Balfourier *et al.* (2007)
**69**	Nyu Bay	Japan	AGG	Balfourier *et al.* (2007)
**70**	Tau-Bugda	Russia[3]	AGG	Balfourier *et al.* (2007)
**71**	Austro Bankut	Austria	AGG	Balfourier *et al.* (2007)
**72**	Apex	Canada	AGG	Balfourier *et al.* (2007)
**73**	Ferrugineum	Russia[3]	AGG	Balfourier *et al.* (2007)
**74**	Nong Da 141	China	AGG	Balfourier *et al.* (2007)
**75**	Songlen	Iraq	CSIRO	Drought tolerant
**76**	Caribo	Germany	AGG	Balfourier *et al.* (2007)
**77**	Minturk	United States	AGG	Balfourier *et al.* (2007)
**78**	Canuck	Canada	AGG	Balfourier *et al.* (2007)
**79**	Limpopo	Zimbabwe	AGG	Balfourier *et al.* (2007)
**80**	Balkan	Yugoslavia	AGG	Balfourier *et al.* (2007)
**81**	Arkas	France	AGG	Balfourier *et al.* (2007)
**82**	Rongotea	New Zealand	AGG	Balfourier *et al.* (2007)
**83**	Buck Buck S	Mexico	AGG	Balfourier *et al.* (2007)
**84**	Emu S	Mexico	AGG	Balfourier *et al.* (2007)
**85**	Horoshiri-Komugi	Japan	AGG	Balfourier *et al.* (2007)
**86**	Benni	United States	AGG	Balfourier *et al.* (2007)
**87**	Katyil	Australia	AGG	Balfourier *et al.* (2007)
**88**	Ogosta	Bulgaria	AGG	Balfourier *et al.* (2007)
**89**	Akadaruma	Japan	AGG	Balfourier *et al.* (2007)
**90**	Bass	Australia	AGG	Balfourier *et al.* (2007)
**91**	Compton	United States	AGG	Balfourier *et al.* (2007)
**92**	Corin	United Kingdom	AGG	Balfourier *et al.* (2007)
**93**	Comet	Australia	AGG	Balfourier *et al.* (2007)
**94**	Opata 85	Mexico	AGG	Balfourier *et al.* (2007)
**95**	Wattines	Germany	AGG	Balfourier *et al.* (2007)
**96**	Turda 81	Romania	AGG	Balfourier *et al.* (2007)
**97**	Recital	France	AGG	Balfourier *et al.* (2007)
**98**	Adular	Germany	AGG	Balfourier *et al.* (2007)
**99**	Granit	Germany	AGG	Balfourier *et al.* (2007)
**100**	Dal'nevostochnaya 10	Russia[3]	AGG	Balfourier *et al.* (2007)
**101**	Corsodor	France	AGG	Balfourier *et al.* (2007)
**102**	3716-1	Bulgaria	AGG	Balfourier *et al.* (2007)
**103**	Mv Ma	Bulgaria	AGG	Balfourier *et al.* (2007)
**104**	Flamura 85	Romania	AGG	Balfourier *et al.* (2007)
**105**	Axona	United Kingdom	AGG	Balfourier *et al.* (2007)

**106**	Genesis	Canada	AGG	
**107**	Hana	Czech Republic	AGG	Balfourier *et al.* (2007)
**108**	Kirac 66	Turkey	AGG	Balfourier *et al.* (2007)
**109**	Dneprovskaya 155	Russia[3]	AGG	Balfourier *et al.* (2007)
**110**	Szoke (GK)	Hungary	AGG	Balfourier *et al.* (2007)
**111**	Grenier	France	AGG	Balfourier *et al.* (2007)
**112**	Sary Bugda	Russia[3]	AGG	
**113**	Renan	France	AGG	Balfourier *et al.* (2007)
**114**	Landrace	Armenia	AGG	
**115**	Apache	France	AGG	Balfourier *et al.* (2007)
**116**	Kraka	Denmark	AGG	Balfourier *et al.* (2007)
**117**	Cadenza	United Kingdom	AGG	Balfourier *et al.* (2007)
**118**	Soor Ghanum	Pakistan	AGG	
**119**	W7984	Mexico	AGG	
**120**	Strubes Dickopf	Germany	AGG	Balfourier *et al.* (2007)
**121**	Bonnie Rock	Australia	InterGrain	WA top 10 cultivar
**122**	Calingiri	Australia	InterGrain	WA top 10 cultivar
**123**	Cobra	Australia	LPB	WA top 10 cultivar
**124**	Corack	Australia	AGT	WA top 10 cultivar
**125**	Justica Cl Plus	Australia	AGT	WA top 10 cultivar
**126**	Mace	Australia	AGT	WA top 10 cultivar; released in 2014
**127**	Magenta	Australia	InterGrain	WA top 10 cultivar
**128**	Stiletto	Australia	ARI	WA top 10 cultivar
**129**	Wyalkatchem	Australia	InterGrain	WA top 10 cultivar; released in 2001
**130**	Yitpi	Australia	ARI	WA top 10 cultivar
**131**	Bremer	Australia	AGT	Recent release
**132**	Harper	Australia	InterGrain	Recent release
**133**	Hydra	Australia	InterGrain	Recent release
**134**	Impress Cl Plus	Australia	InterGrain	Recent release
**135**	Supreme	Australia	InterGrain	Recent release; Narayanan *et al.* (2014)
**136**	Trojan	Australia	LPB	Recent release
**137**	Zen	Australia	InterGrain	Recent release
**138**	Drysdale	Australia	CSIRO	Saradadevi *et al.* (2014)[5]; released in 2002
**139**	Grenade Cl Plus	Australia	AGT	Recent release; drought tolerant
**140**	Igw-3119	Australia	CSIRO	Saradadevi *et al.* (2014)
**141**	Igw-3262	Australia	CSIRO	Saradadevi *et al.* (2014)
**142**	Scepter	Australia	AGT	Recent release; drought tolerant
**143**	Beyaz Tir	Turkey	AWCC	
**144**	Ciano 67	Mexico	AWCC	
**145**	Downy	United States	AWCC	
**146**	Gleennson 81	Mexico	AWCC	
**147**	Hobbit Sib	United Kingdom	AWCC	
**148**	Hongmang	China	AWCC	
**149**	IBIS	Denmark	AWCC	
**150**	Kauz Dwarf	Mexico	AWCC	
**151**	Kauz S	Mexico	AWCC	
**152**	Maris Templar	United Kingdom	AWCC	
**153**	PI 74494	Russia[3]	AWCC	
**154**	Seri 82	Mexico	AWCC	Narayanan *et al.* (2014)
**155**	Sonora 64	Mexico	AWCC	Narayanan *et al.* (2014)
**156**	Sportsman	United Kingdom	AWCC	
**157**	Tincurrin	Australia	AWCC	
**158**	Yecora	Mexico	AWCC	
**159**	Kauz Tall	Mexico	AWCC	
**160**	AMC 71	Iraq	CSIRO	Drought tolerant
**161**	Condor	Australia	CSIRO	Released in 1997

**162**	Cranbrook	Australia	CSIRO	
**163**	Egawildy	Australia	CSIRO	Balfourier *et al.* (2007)
**164**	Excalibar	Australia	CSIRO	
**165**	Frame	Australia	CSIRO	Released in 1994
**166**	Gamenya B	Australia	CSIRO	Balfourier *et al.* (2007)
**167**	gladius	Australia	CSIRO	Released in 2007
**168**	Halberd	Australia	CSIRO	Released in 1969
**169**	Hartog	Australia	CSIRO	Narayanan *et al.* (2014)
**170**	Heron	Australia	CSIRO	Released in 1958
**171**	Janz	Australia	CSIRO	Released in 1989
**172**	Kalannie	Australia	CSIRO	
**173**	Kennedy	Australia	CSIRO	
**174**	Krichauff	Australia	CSIRO	Released in 1997
**175**	Lincoln	Australia	CSIRO	More tillers
**176**	Livingston	Australia	CSIRO	
**177**	Machete	Australia	CSIRO	
**178**	Magsil 30	Australia	CSIRO	
**179**	RAC 875	Australia	CSIRO	
**180**	SB 034	Mexico	CSIRO	
**181**	SB 072	Mexico	CSIRO	
**182**	Silverstar	Australia	CSIRO	
**183**	Sunco	Australia	CSIRO	
**184**	Warigal	Australia	CSIRO	

Notes:
[1] County of origin (number of genotypes): Australia (55), Mexico (18), Nepal (13), Russia (10), Canada (9), United Kingdom (9), United States (8), France (6), Germany (5), Bulgaria (3), China (3), Japan (3), Kenya (3), Turkey (3), Argentina (2), Austria (2), Denmark (2), Finland (2), Hungary (2), Iraq (2), Italy (2), New Zealand (2), Pakistan (2), Poland (2), Portugal (2), Romania (2), Tunisia (2), Algeria (1), Armenia (1), Brazil (1), Czech Republic (1), Netherlands (1), South Africa (1), South Korea (1), Spain (1), Yugoslavia (1), Zimbabwe (1). Continent of origin (number of genotypes): Africa (10), Asia (30), Europe (51), North America (35), Oceania (57) and South America (1).

[2] Balfourier F, Roussel V, Strelchenko P, Exbrayat-Vinson F, Sourdille P, Boutet G, Koenig J, Ravel C, Mitrofanova O, Beckert M, Charmet G (2007) A worldwide bread wheat core collection arrayed in a 384-well plate. Theoretical and Applied Genetics. 114(7): 1265−75.

[3] Narayanan S, Mohan A, Gill KS, Prasad PV (2014) Variability of root traits in spring wheat germplasm. PLoS One 9: e100317.

[4] The country of origin of these nine genotypes originally recorded as “Former Soviet Union” were combined with “Russia” in this study.

[5] Saradadevi R, Bramley H, Siddique KH, Edwards E, Palta JA (2014) Contrasting stomatal regulation and leaf ABA concentrations in wheat genotypes when split root systems were exposed to terminal drought. Field Crops Research. 165: 5−14.

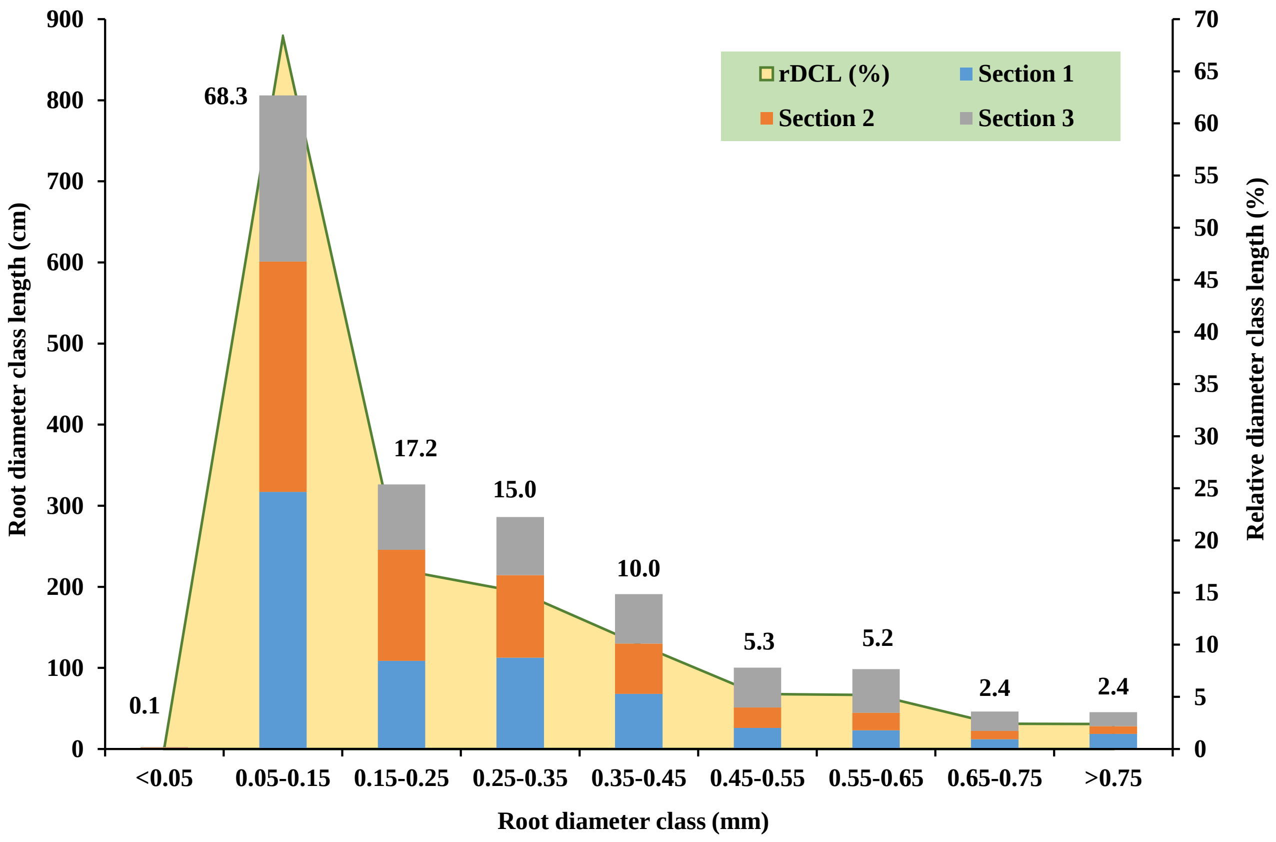

Fig. S1. Root diameter class length (DCL, cm) in sections and relative diameter class length (rDCL, %) among 184 wheat genotypes grown in a semi-hydroponic phenotyping platform 35 days after transplanting. Percentage values for rDCL in each diameter class are plotted on the secondary axis. Mean DCL values in each root section are presented with SEs for the total root length in the respective root diameter class.

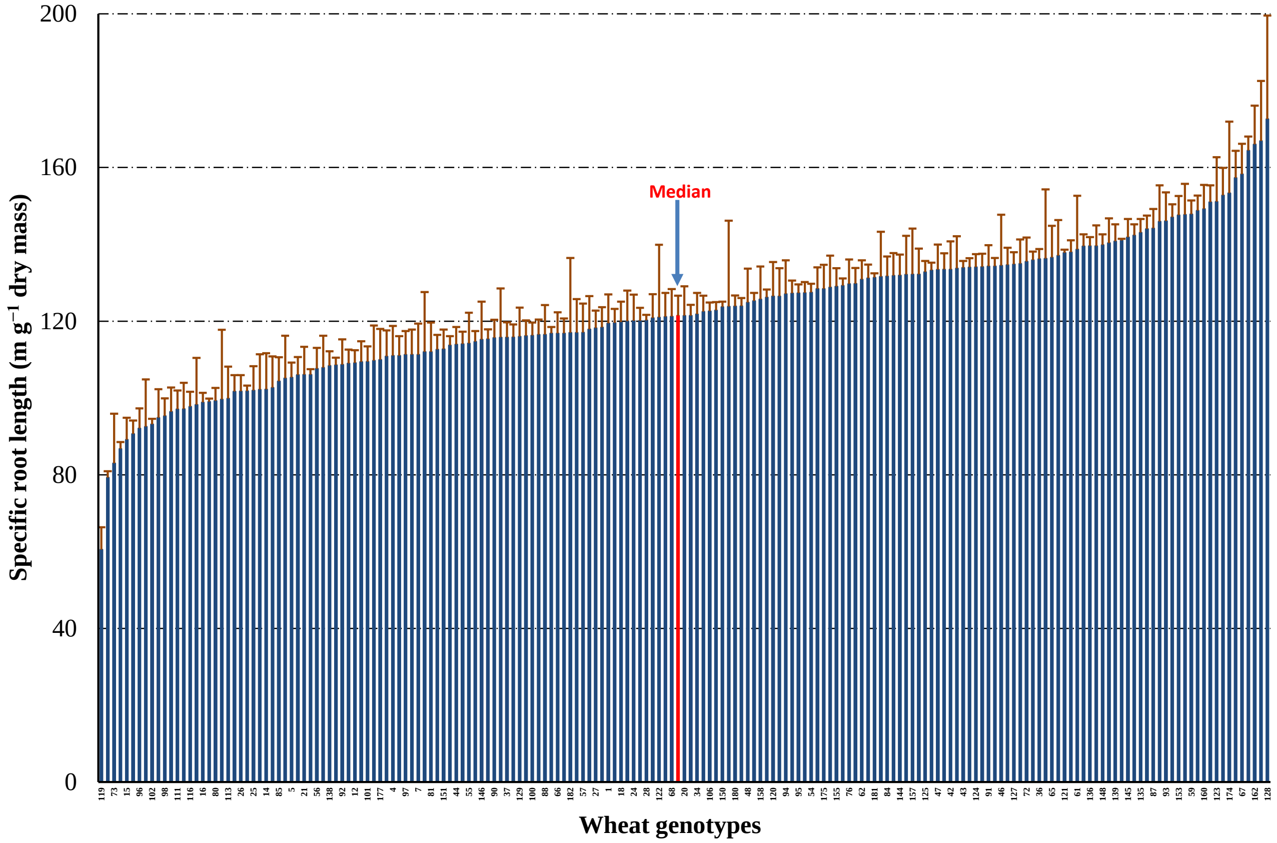

Fig. S2. Phenotypic variation in specific root length (SRL) among 184 wheat genotypes grown in a semi-hydroponic phenotyping platform 35 days after transplanting. Data were plotted from the lowest to the highest SRL values. The median value for all genotypes is presented (red bar).

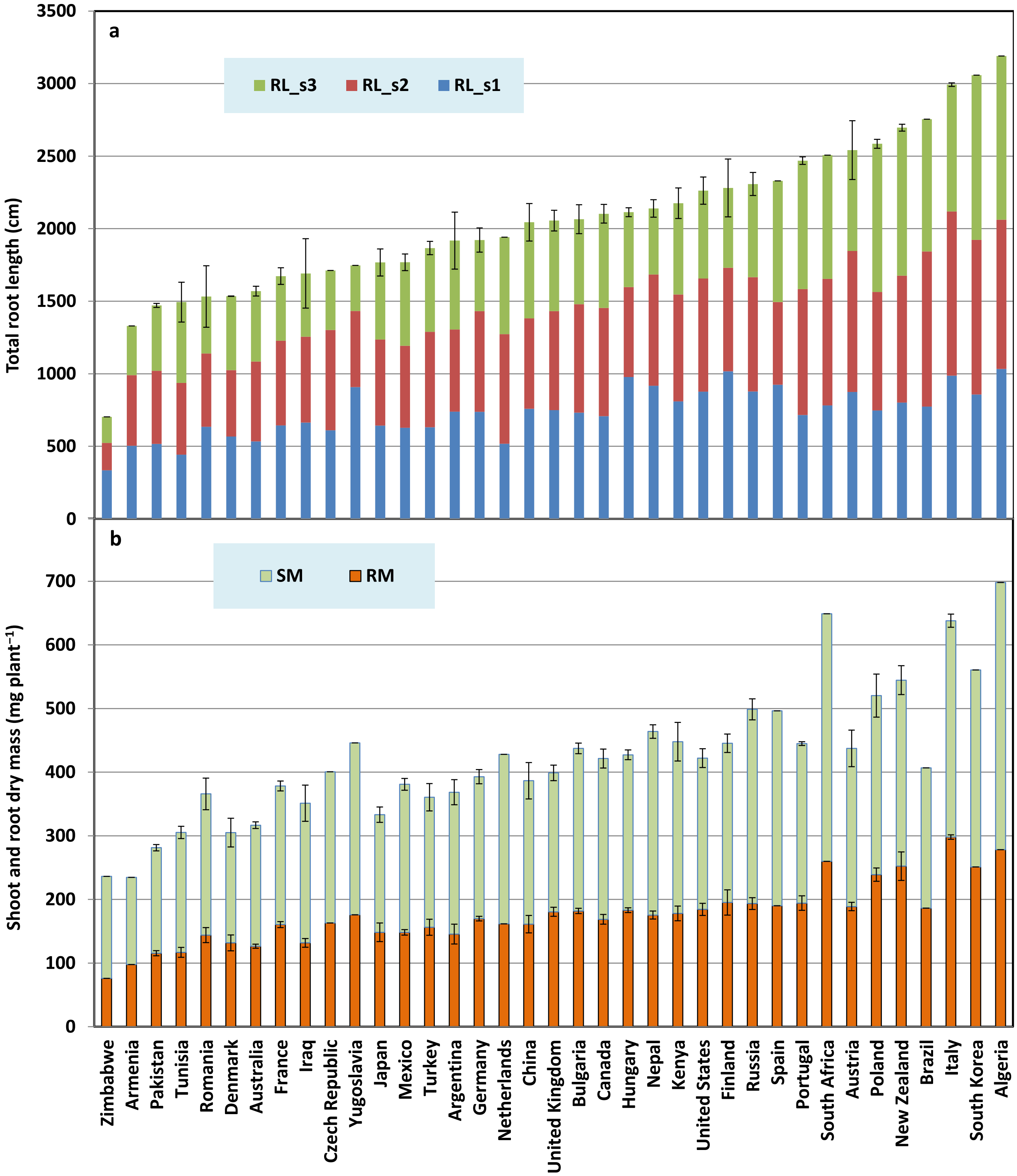

Fig. S3 Variation among the 37 countries of origin in (a) total root length, (b) shoot and root dry mass (SM and RM, respectively) in 184 wheat genotypes grown in a semi-hydroponic phenotyping platform 35 days after transplanting. Root data are the means for each country. Total root length in section 1 (RL_s1, 0–20 cm), section 2 (RL_s2, 20–40 cm), and section 3 (RL_s3, 40–110 cm) ± SE of total root length of all sections is presented. Country names are ordered by the total root length value. The number of genotypes in each country varied and ranged from one to 55 (see Table S3).

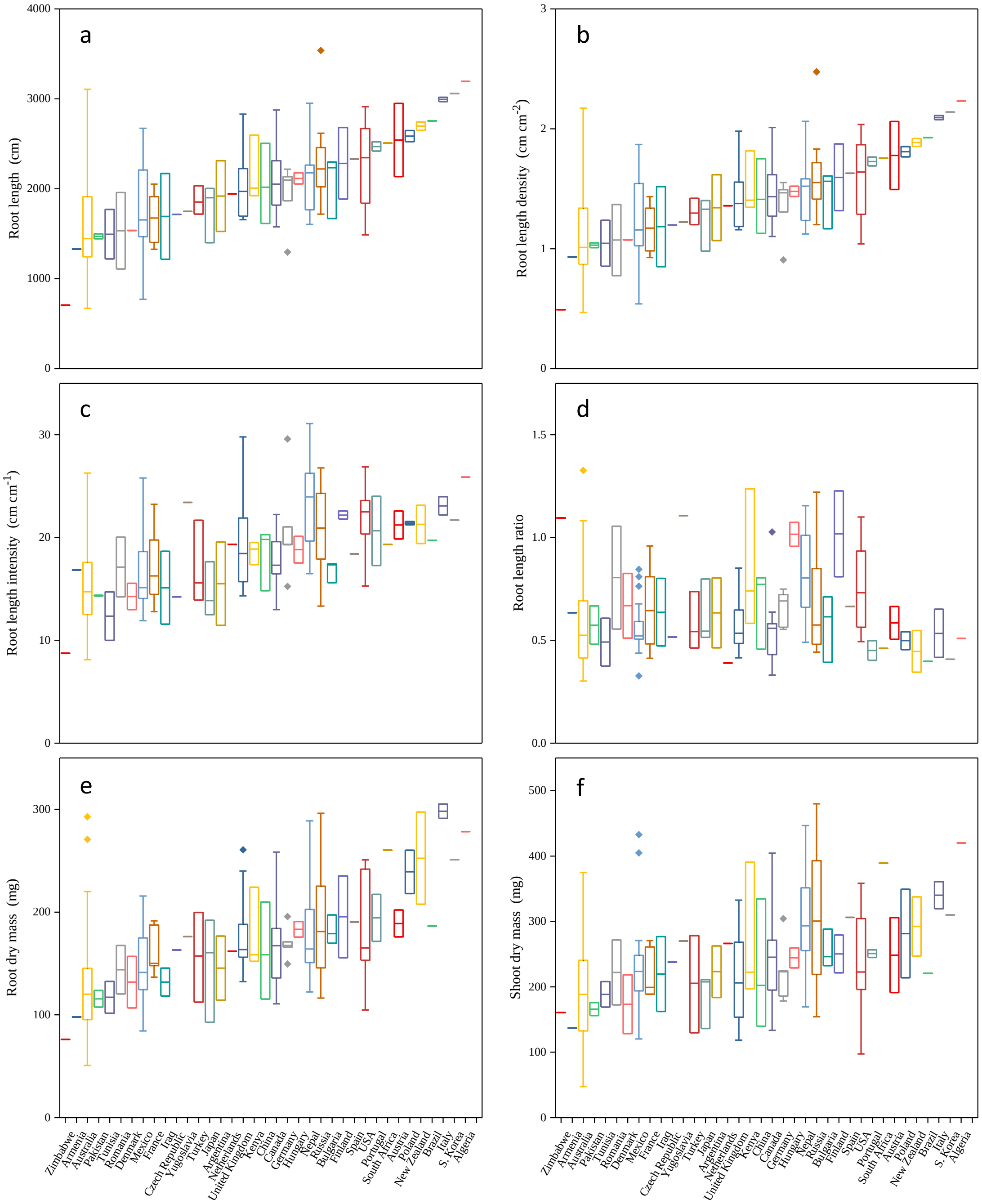

Fig. S4 Variation among the 37 countries of origin in (a) total root length, (b), root length density, (c) root length intensity, (d) root length ratio, (e) root dry mass, and (f) shoot dry mass in 184 wheat genotypes grown in a semi-hydroponic phenotyping platform 35 days after transplanting. Country names are ordered by the median values of root length from least to most. The boxplots were confined to the first and third quartile with the middle lines being the median. The number of genotypes in each country varied and ranged from one to 55 (see Table S3).

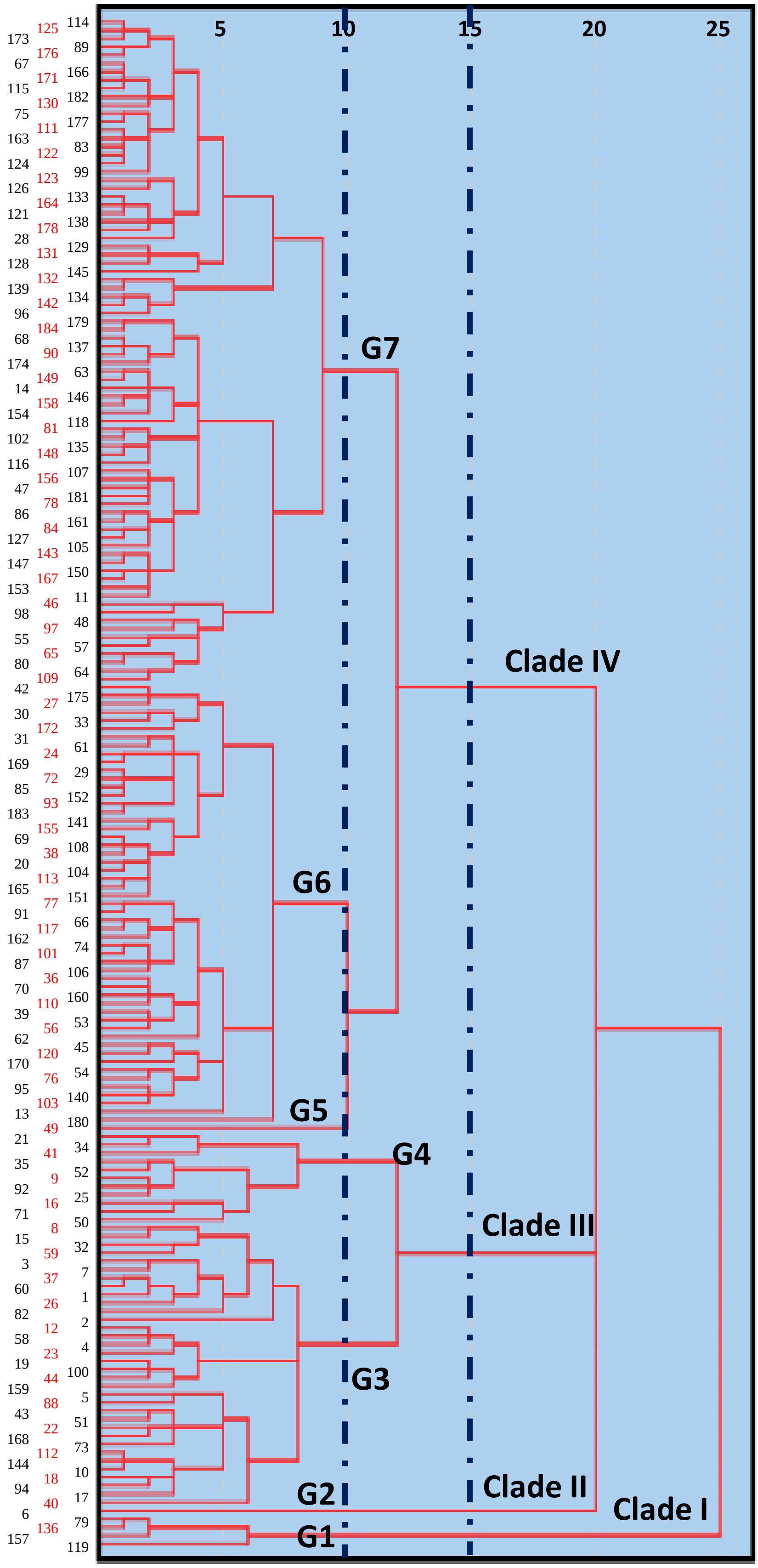

Fig. S5. Dendrogram of agglomerative hierarchical clustering (AHC) using the average linkage method with squared Euclidean distance as the interval measurement on 19 selected root traits with CVs ≥0.25. The 184 wheat genotypes were assigned to one of four general clades (Clade I, II, III or IV) at a rescaled distance of 15 (left dashed line) containing seven groups (G1 to G7) at a rescaled distance of 10 (right dashed line). See Table S3 for a list of the 184 wheat genotypes used in this study.
